# Supplementary figures and images for: Alzheimer’s disease linked Aβ42 exerts product feedback inhibition on γ-secretase impairing downstream cell signaling
Source: eLife. 2024 Jul 19;12:RP90690. doi: 10.7554/eLife.90690 (PMC11259434; doi:10.7554/eLife.90690)

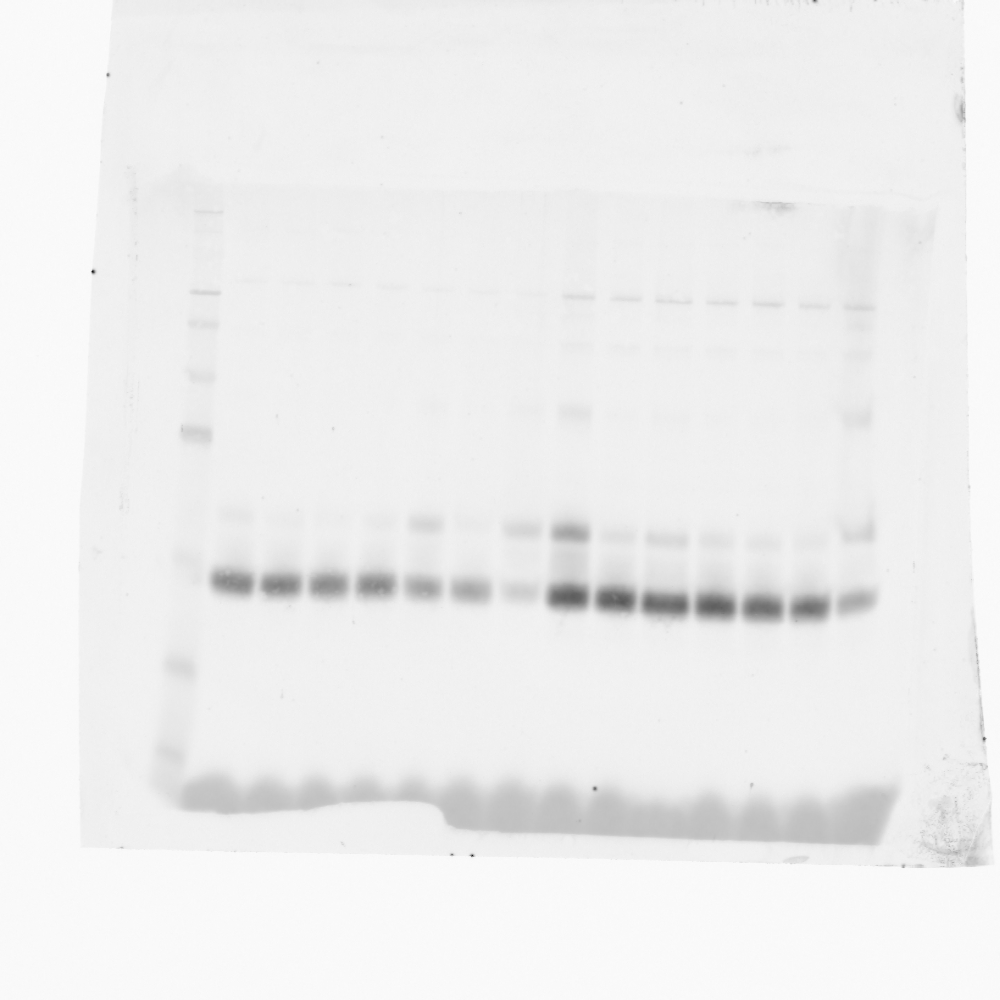

Supplement: Figure 1—source data 1. [file elife-90690-fig1-data1.zip › Figure 1 Source Data 1_org.gel]

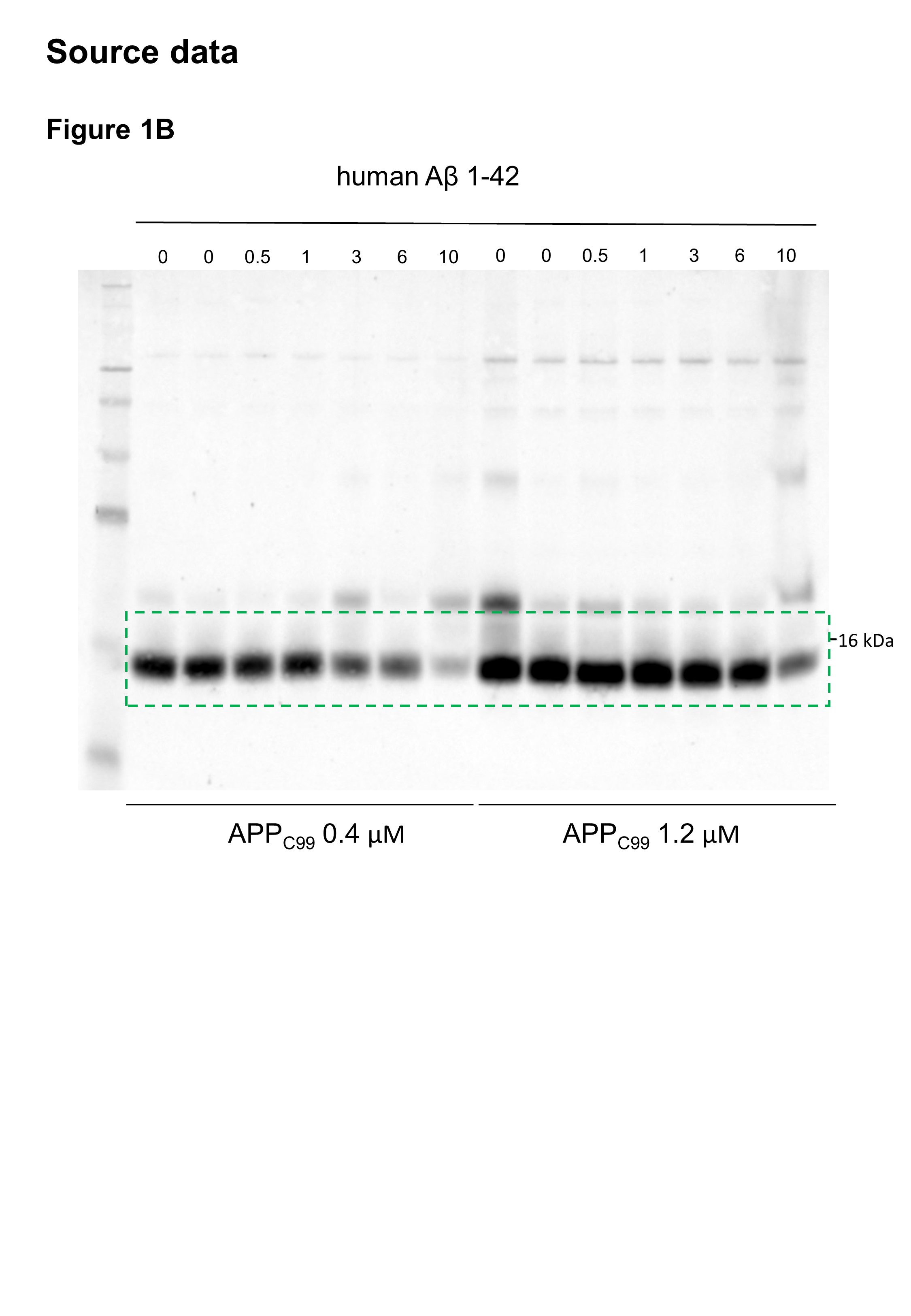

Supplement: Figure 1—source data 1. [file elife-90690-fig1-data1.zip › Figure 1 Source Data 1_panel B.JPG]

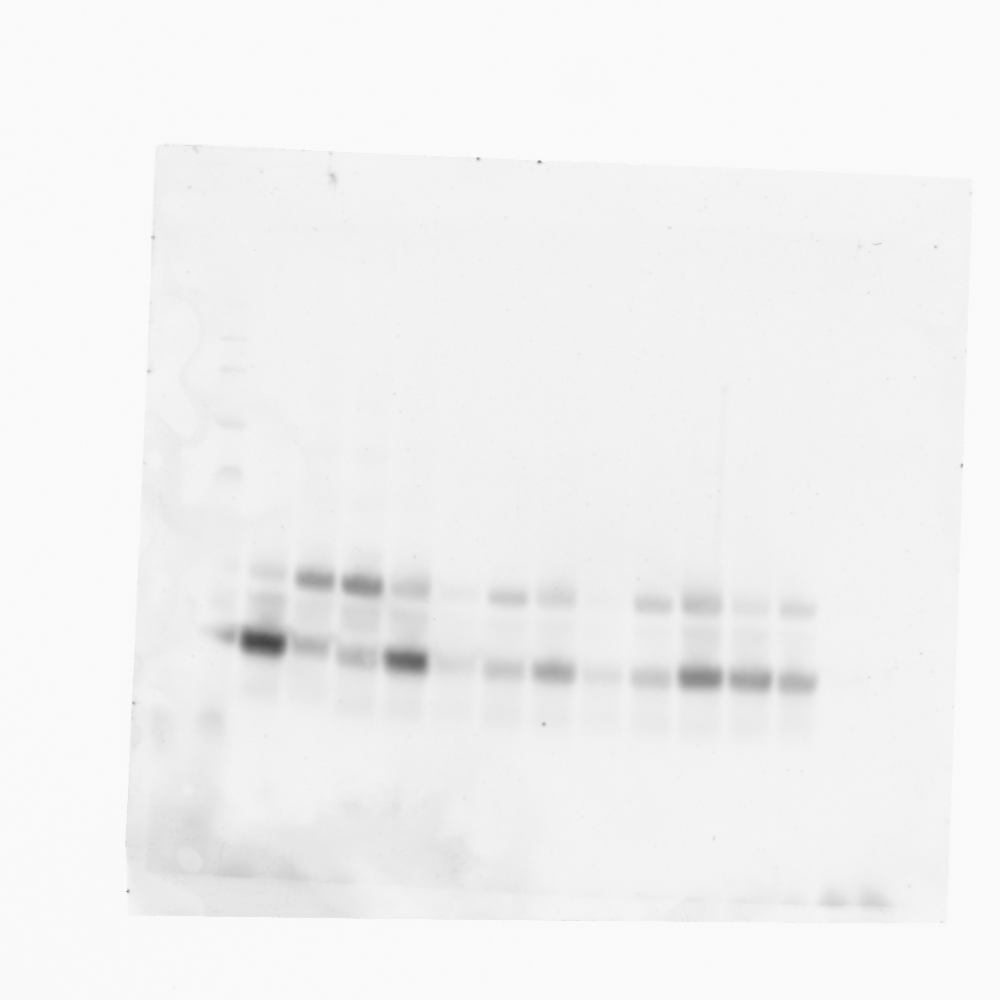

Supplement: Figure 1—source data 1. [file elife-90690-fig1-data1.zip › Figure 1 Source Data 2_org.gel]

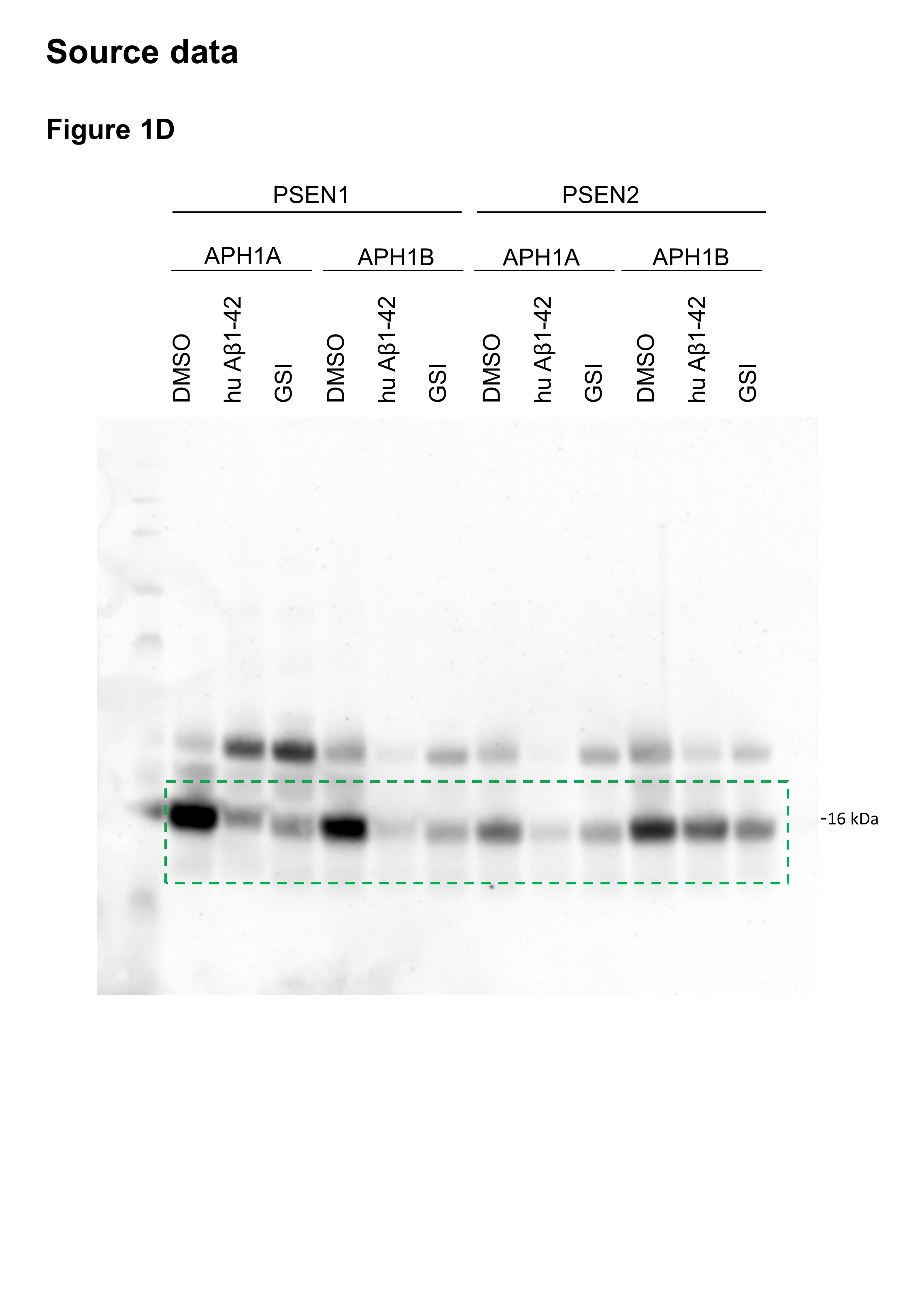

Supplement: Figure 1—source data 1. [file elife-90690-fig1-data1.zip › Figure 1 Source Data 2_panel D.JPG]

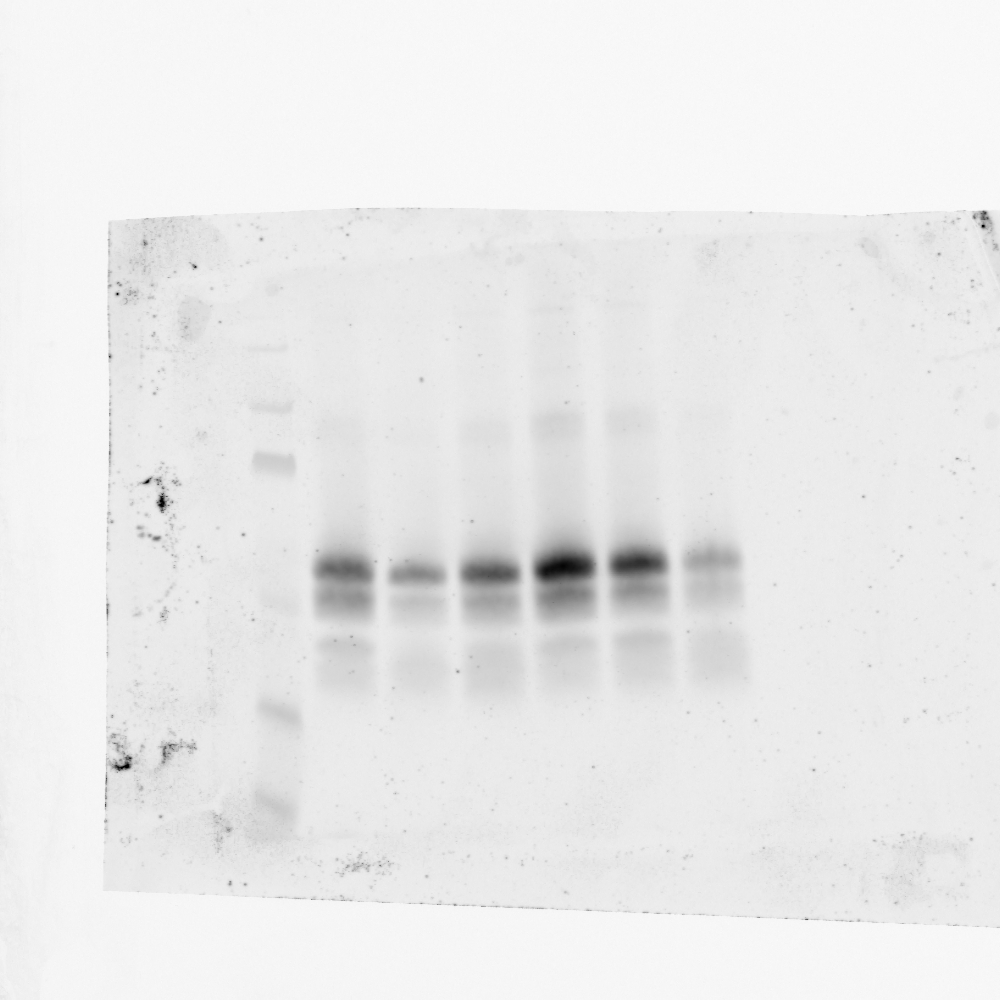

Supplement: Figure 1—source data 1. [file elife-90690-fig1-data1.zip › Figure 1 Source Data 3_org.gel]

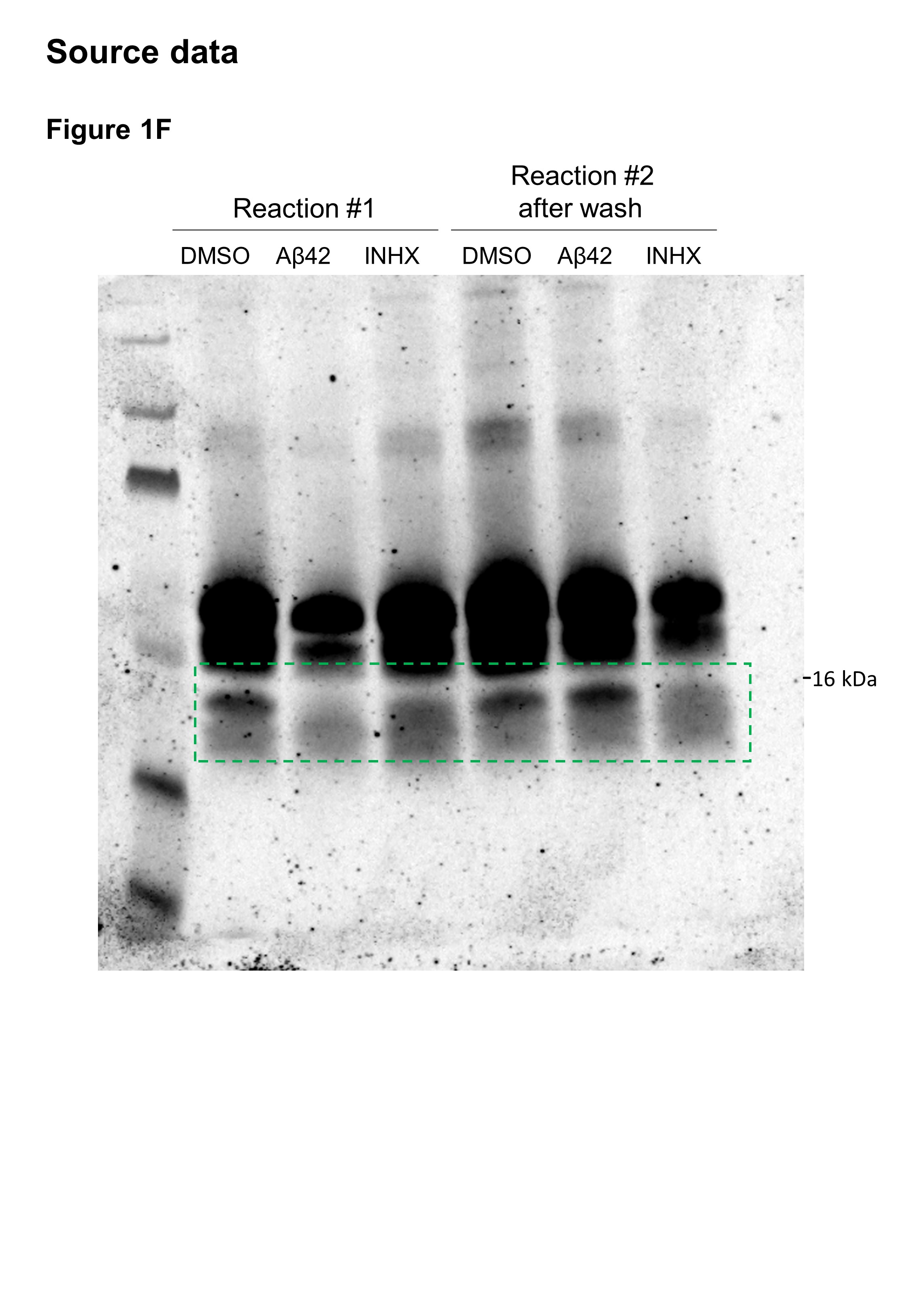

Supplement: Figure 1—source data 1. [file elife-90690-fig1-data1.zip › Figure 1 Source Data 3_panel F.JPG]

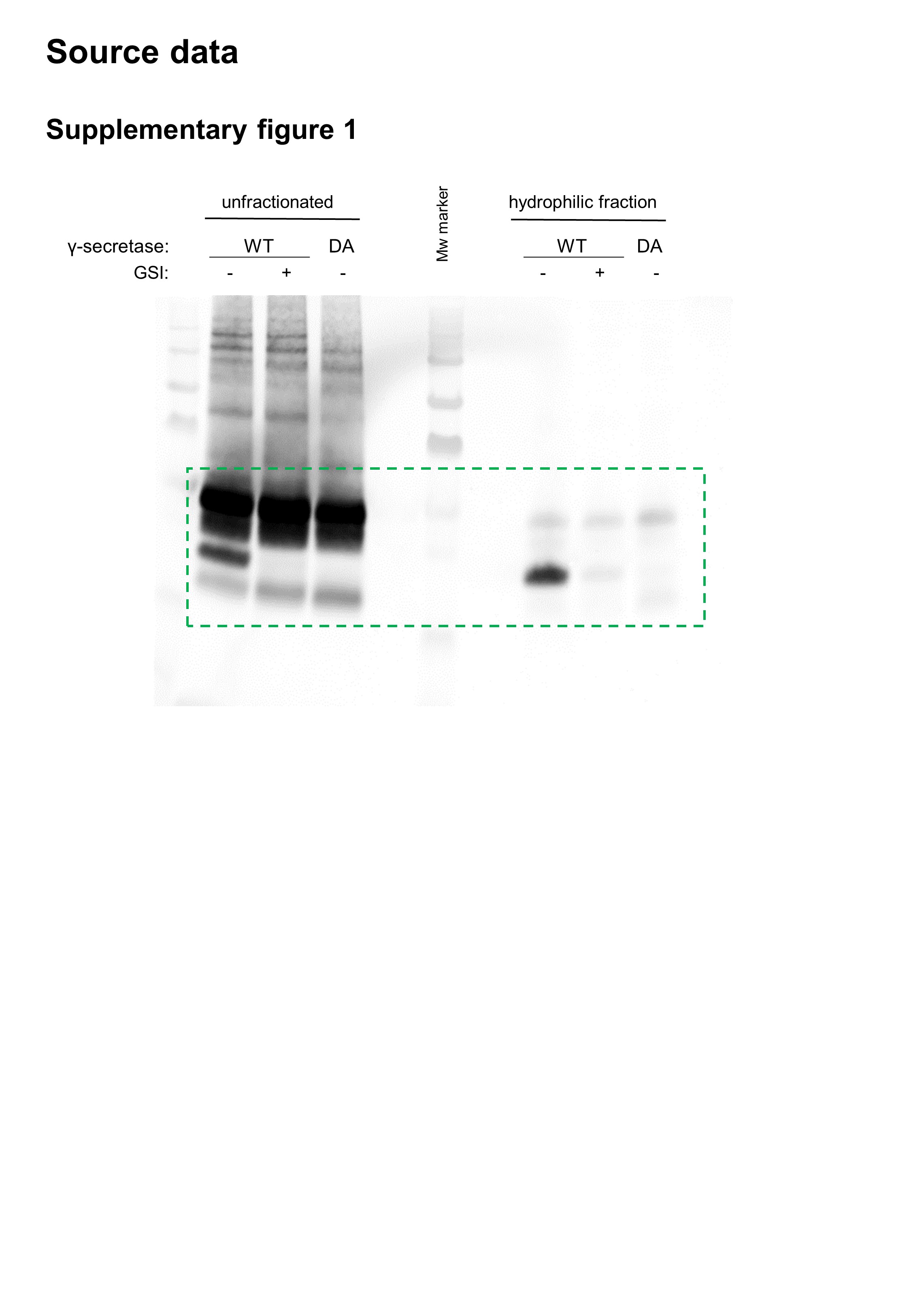

Supplement: Figure 1—figure supplement 1—source data 1. [file elife-90690-fig1-figsupp1-data1.zip › Figure 1 Source Data 4.JPG]

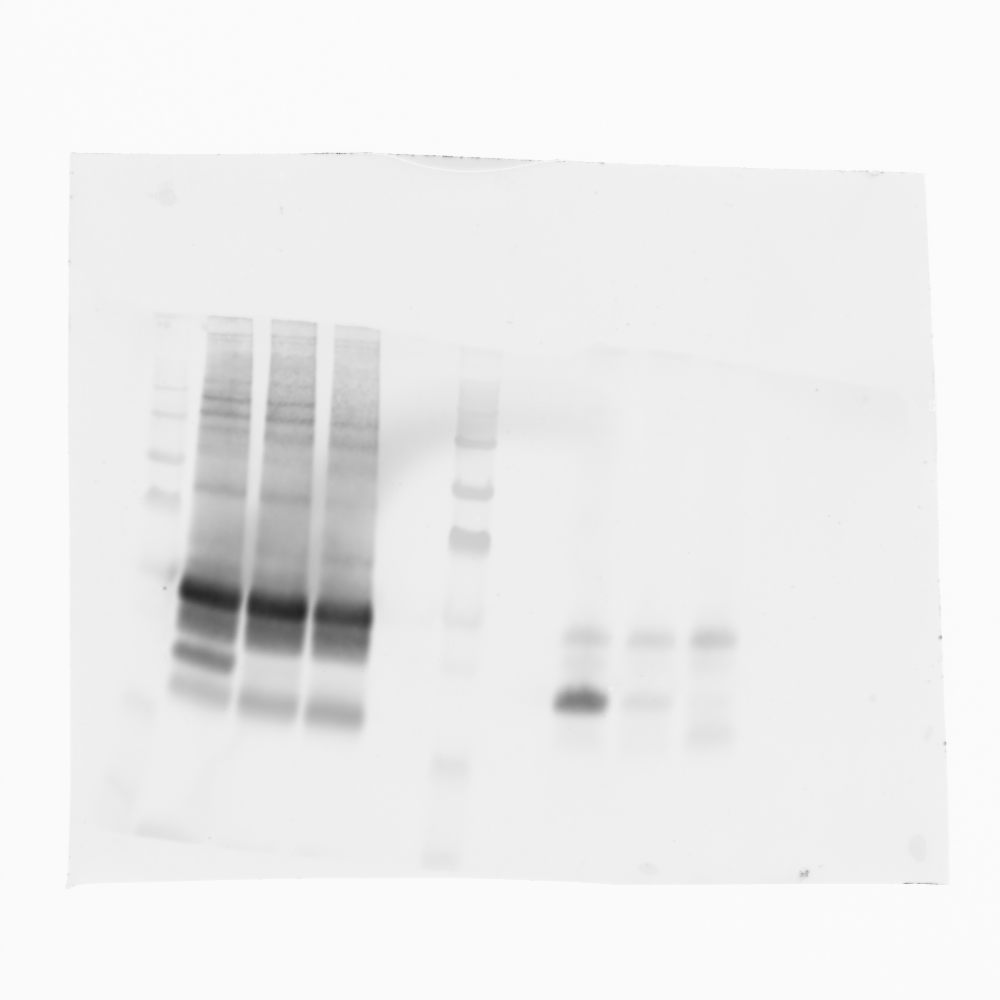

Supplement: Figure 1—figure supplement 1—source data 1. [file elife-90690-fig1-figsupp1-data1.zip › Figure 1 Source Data 4_org.gel]

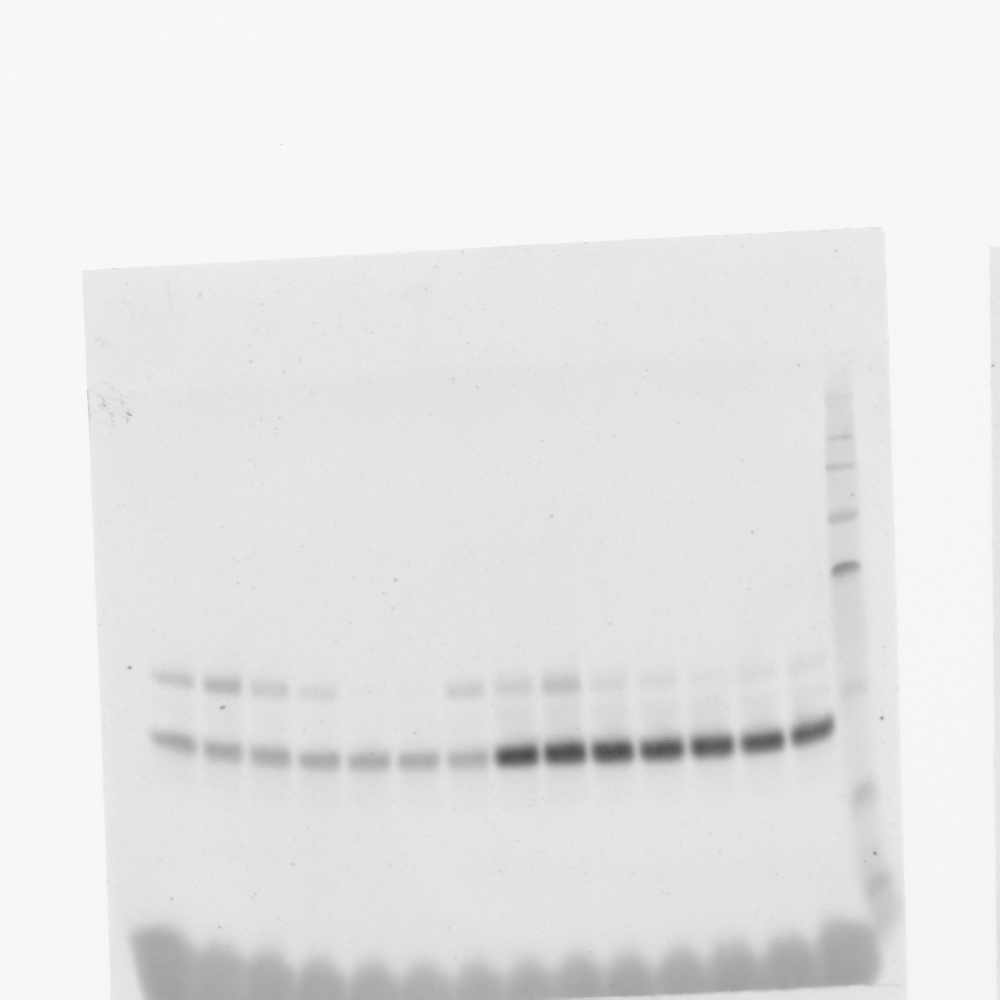

Supplement: Figure 2—source data 1. [file elife-90690-fig2-data1.zip › Figure 2 Source Data 1_org.gel]

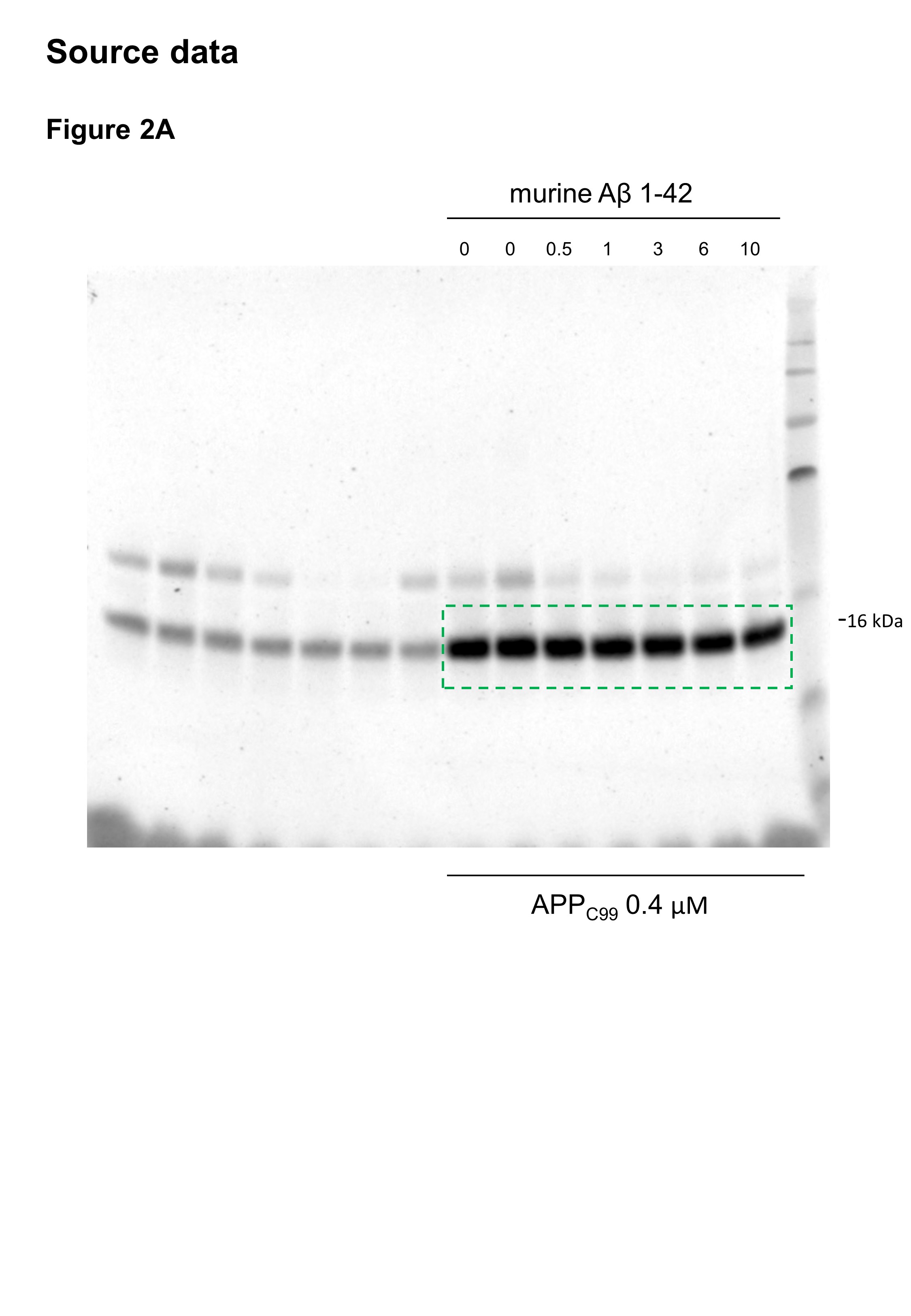

Supplement: Figure 2—source data 1. [file elife-90690-fig2-data1.zip › Figure 2 Source Data 1_panel A.JPG]

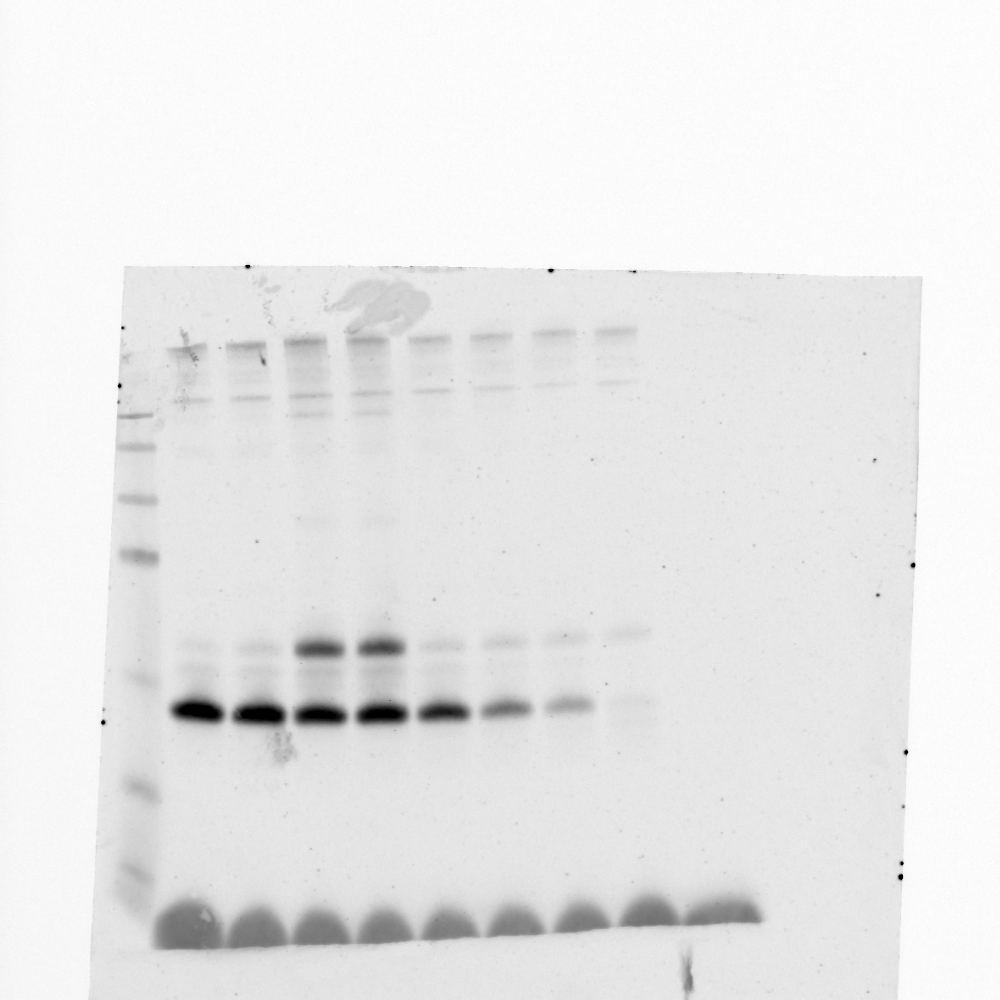

Supplement: Figure 2—source data 1. [file elife-90690-fig2-data1.zip › Figure 2 Source Data 2_org 1.gel]

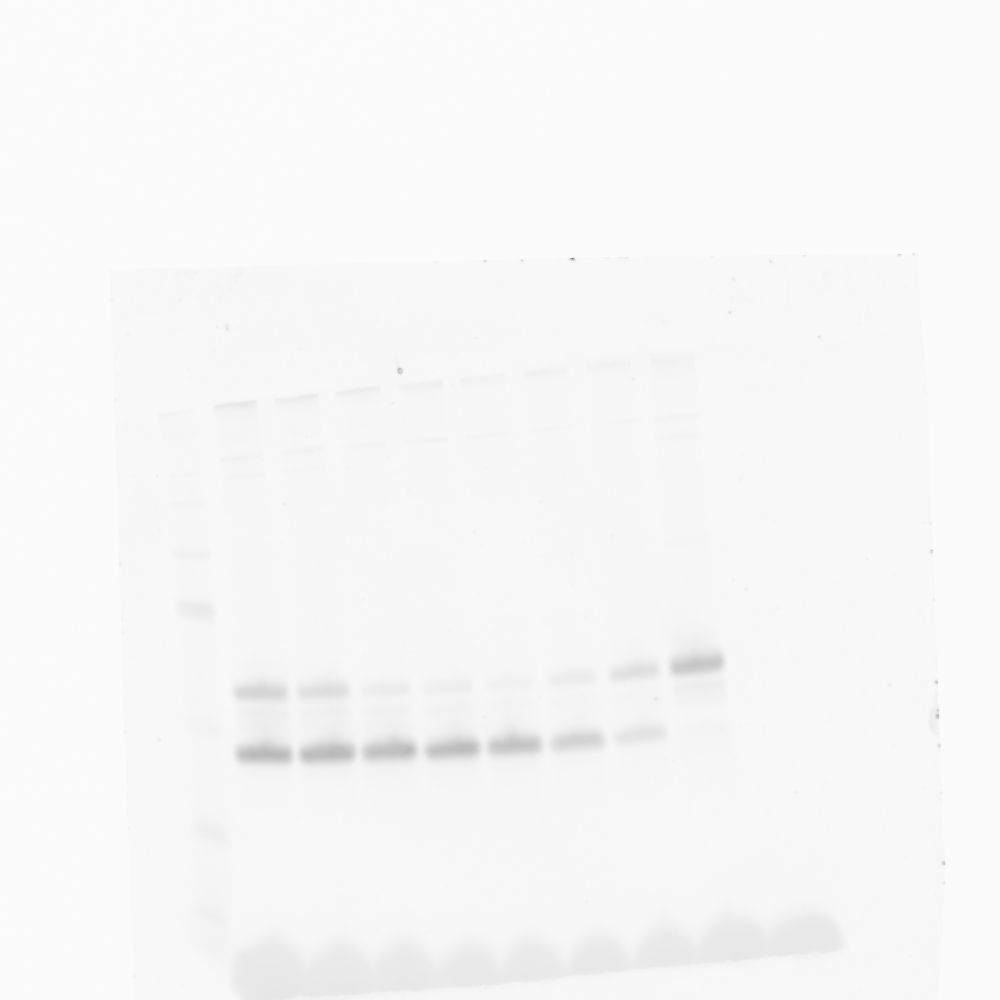

Supplement: Figure 2—source data 1. [file elife-90690-fig2-data1.zip › Figure 2 Source Data 2_org 2.gel]

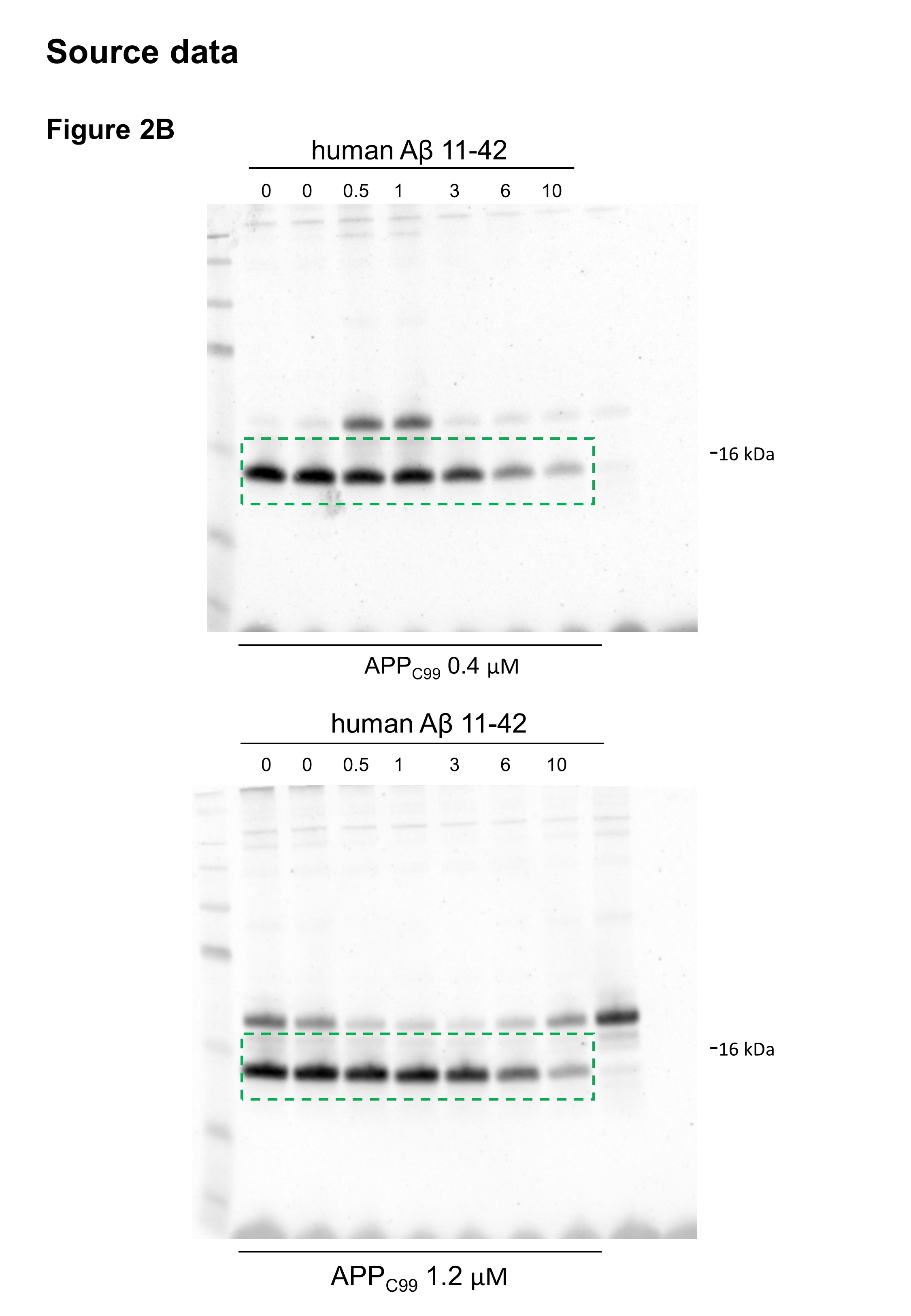

Supplement: Figure 2—source data 1. [file elife-90690-fig2-data1.zip › Figure 2 Source Data 2_panel B.JPG]

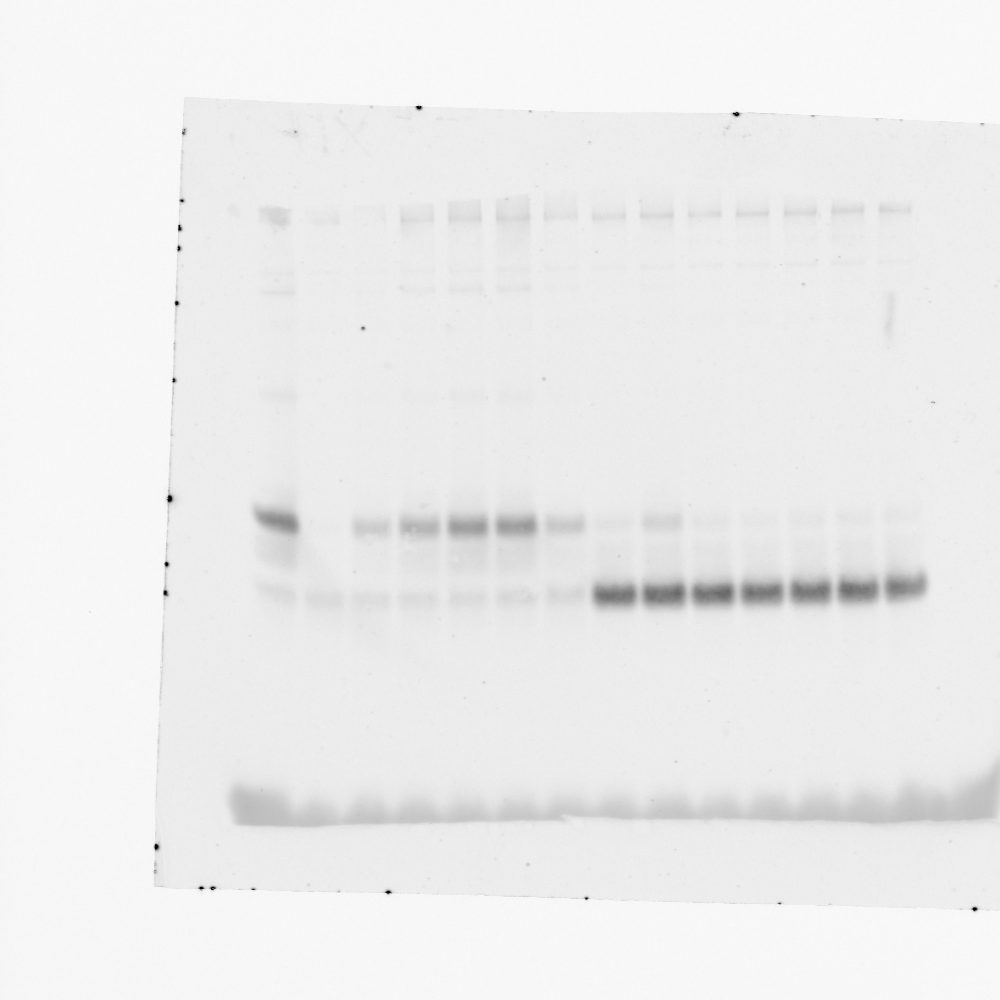

Supplement: Figure 2—source data 1. [file elife-90690-fig2-data1.zip › Figure 2 Source Data 3_org 1.gel]

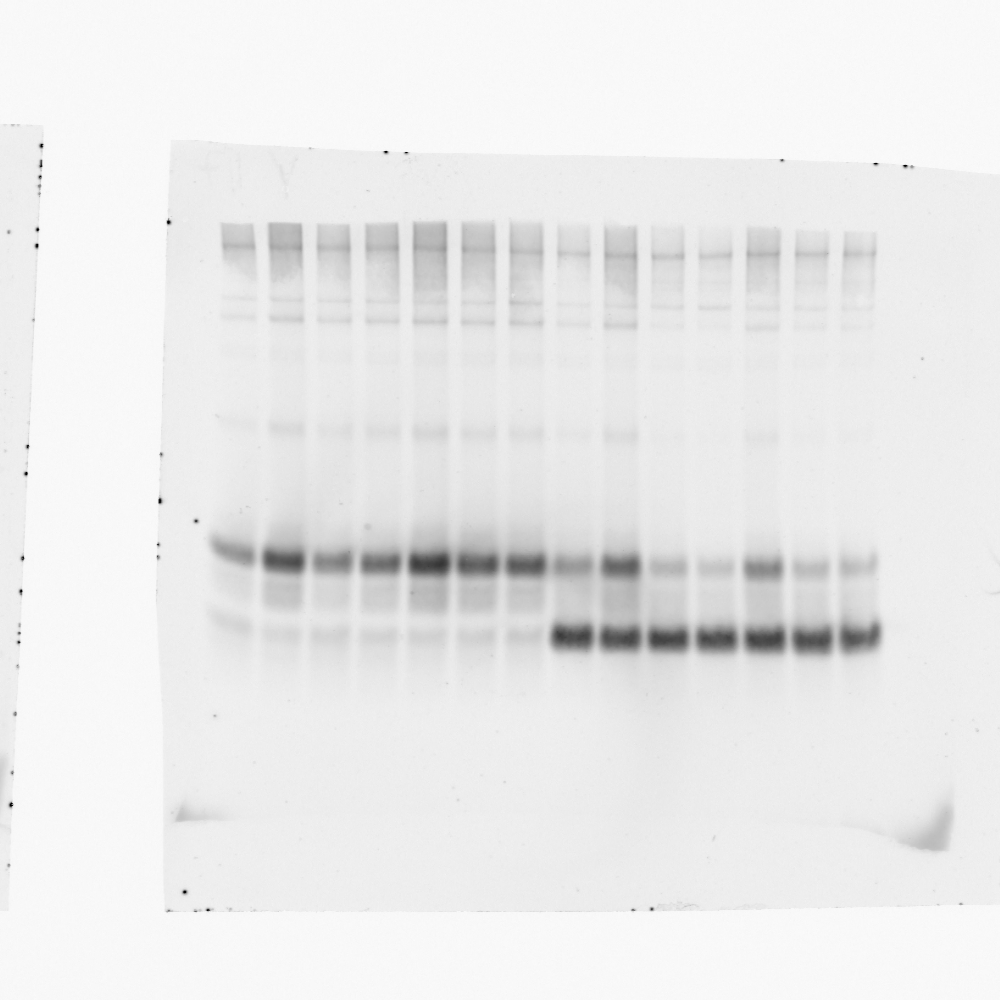

Supplement: Figure 2—source data 1. [file elife-90690-fig2-data1.zip › Figure 2 Source Data 3_org 2.gel]

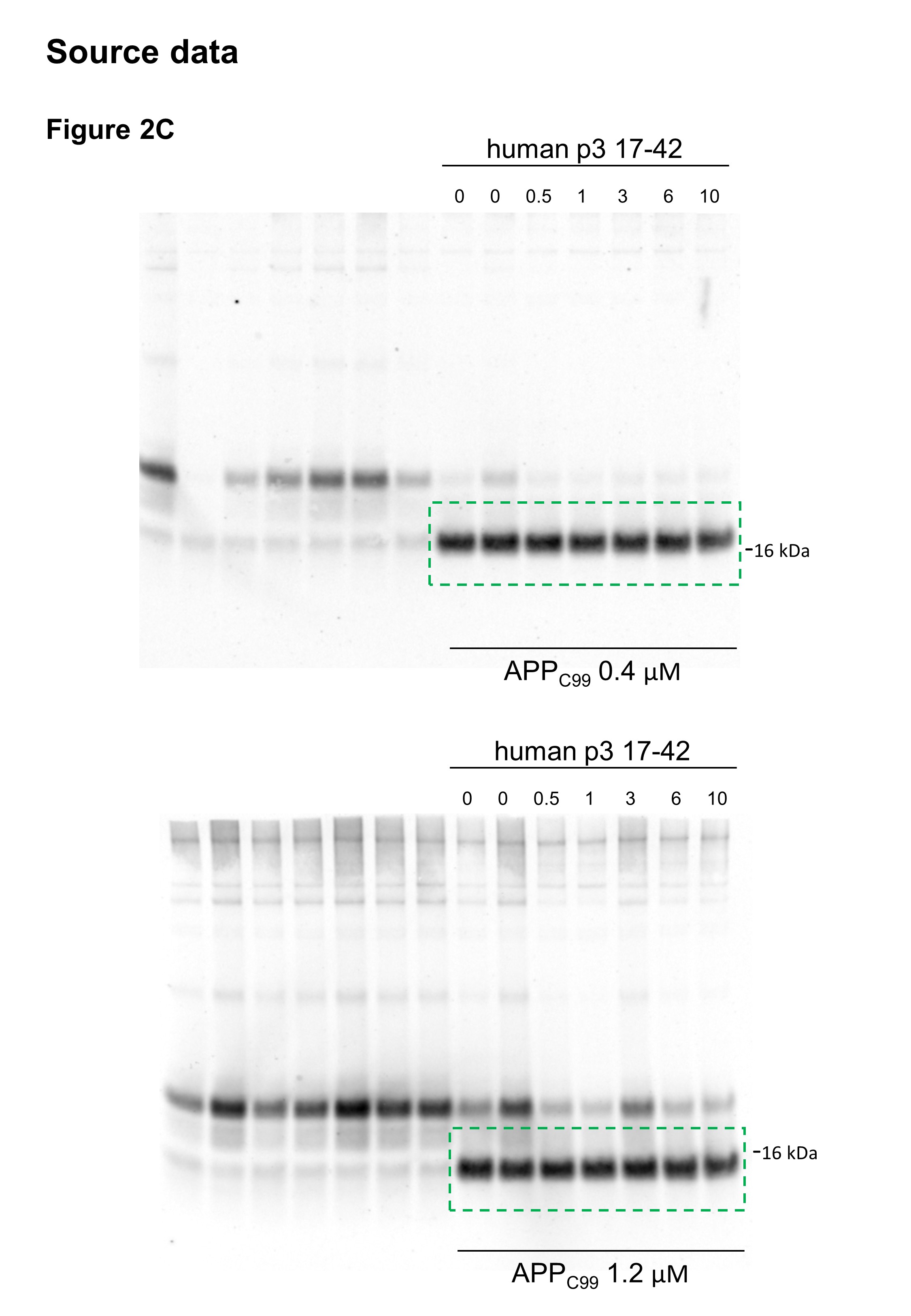

Supplement: Figure 2—source data 1. [file elife-90690-fig2-data1.zip › Figure 2 Source Data 3_panel C.JPG]

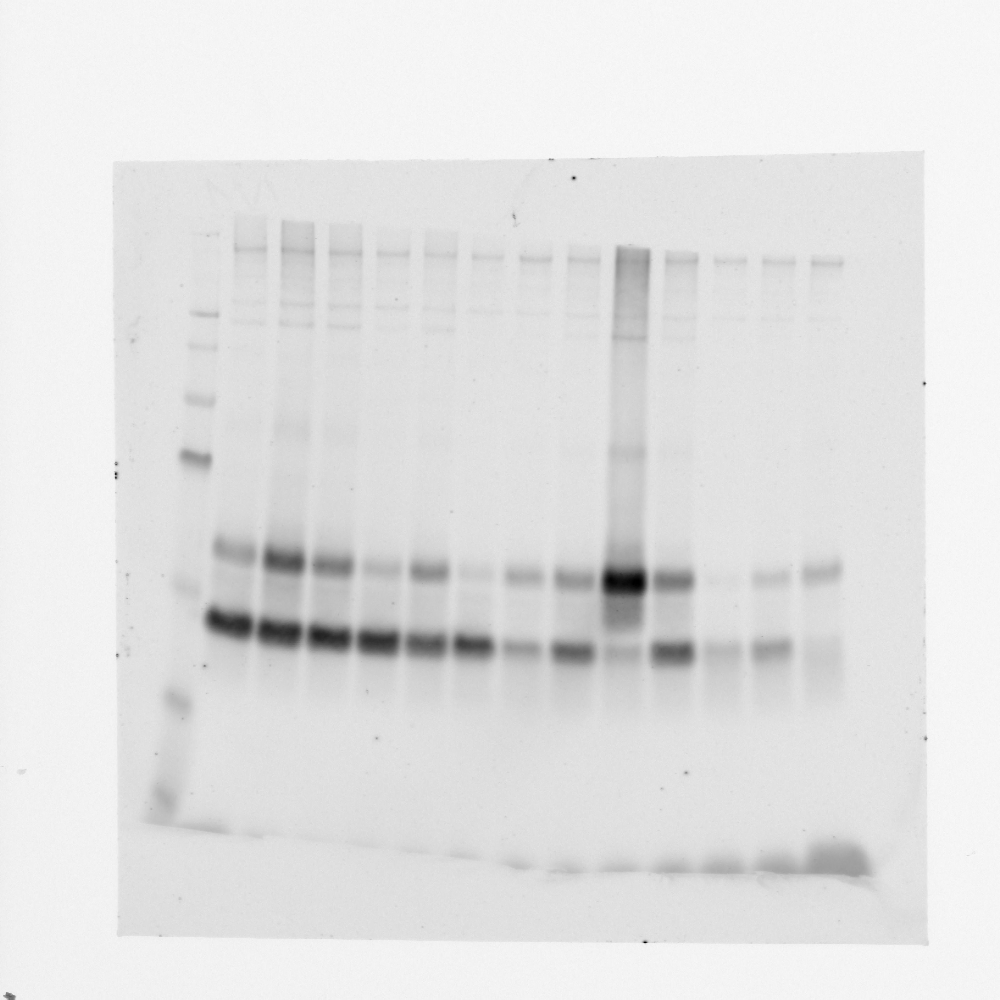

Supplement: Figure 2—source data 1. [file elife-90690-fig2-data1.zip › Figure 2 Source Data 4_org 1.gel]

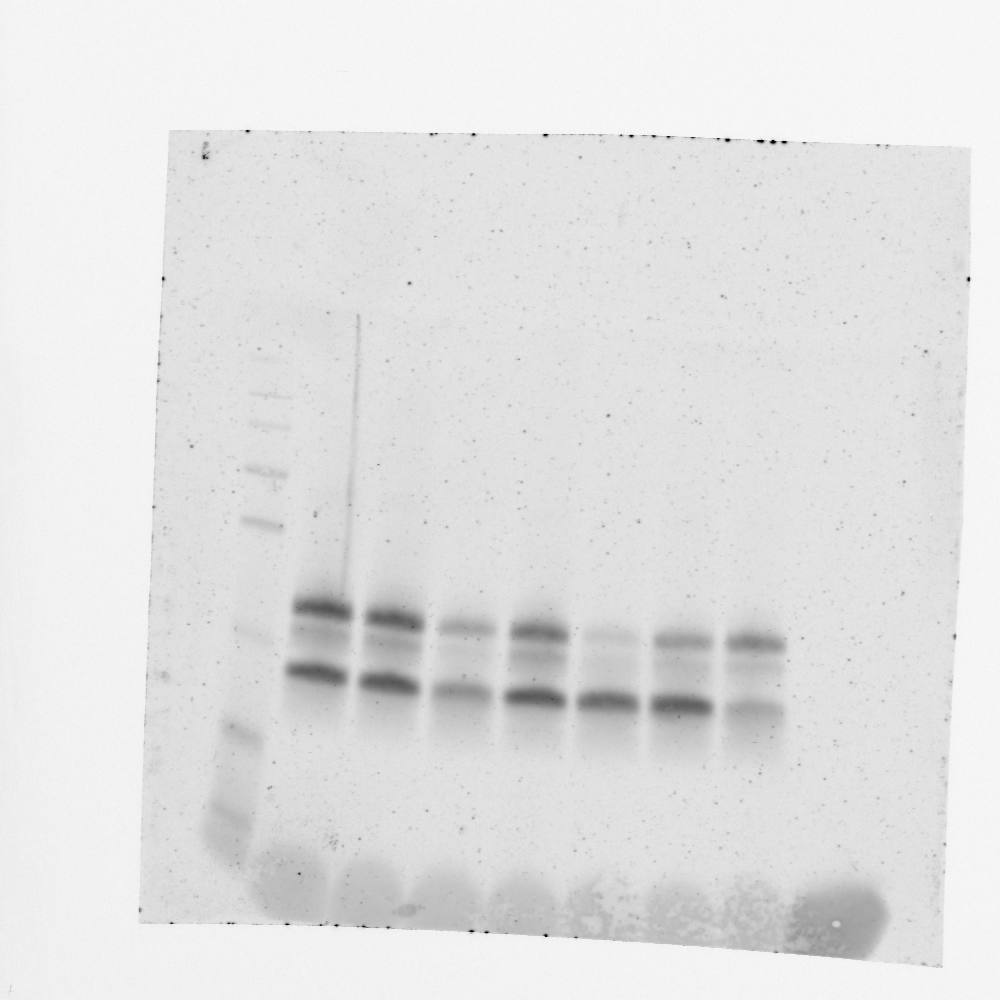

Supplement: Figure 2—source data 1. [file elife-90690-fig2-data1.zip › Figure 2 Source Data 4_org 2.gel]

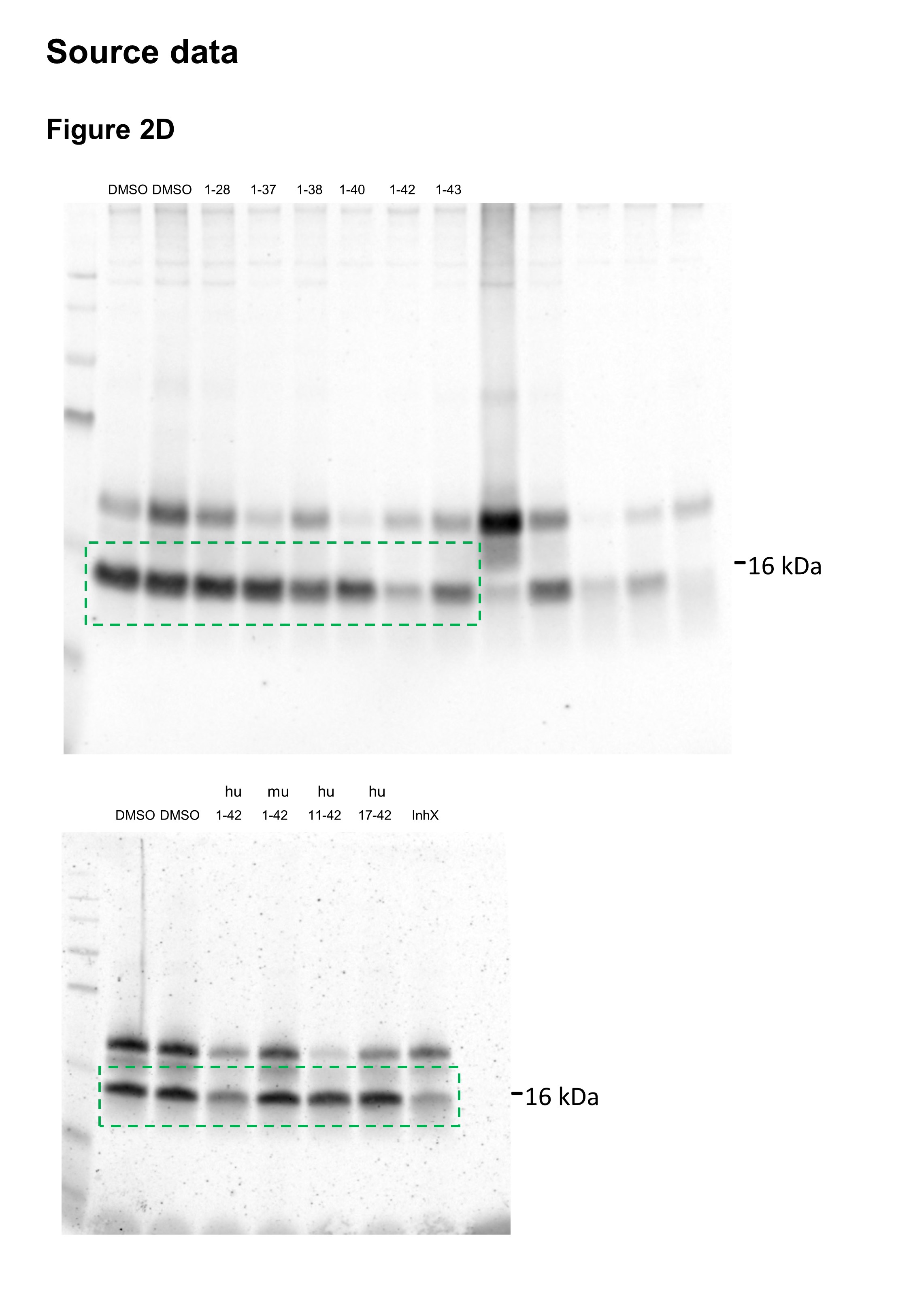

Supplement: Figure 2—source data 1. [file elife-90690-fig2-data1.zip › Figure 2 Source Data 4_panel D.JPG]

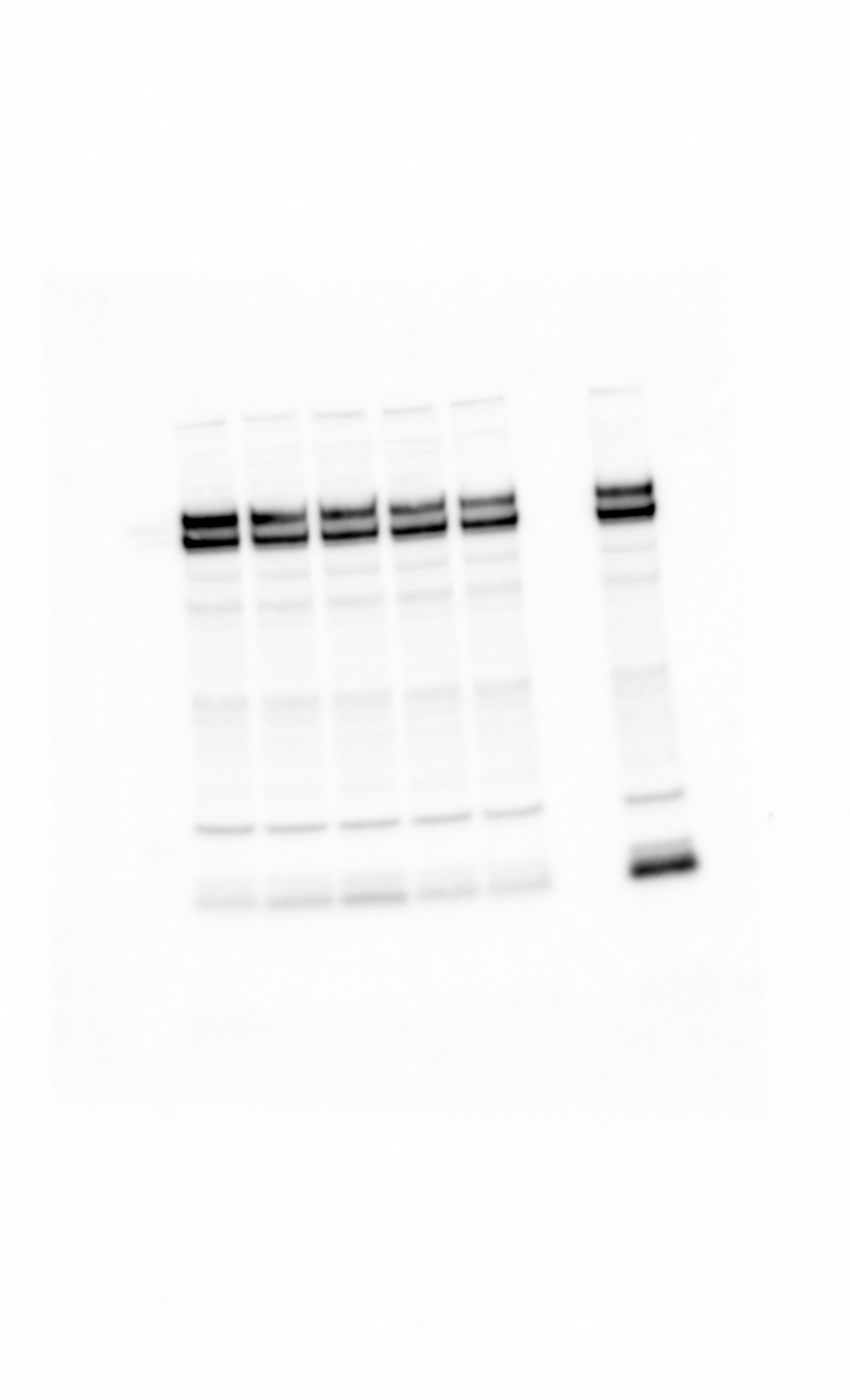

Supplement: Figure 3—source data 1. [file elife-90690-fig3-data1.zip › Figure 3 Source Data 1_org.gel]

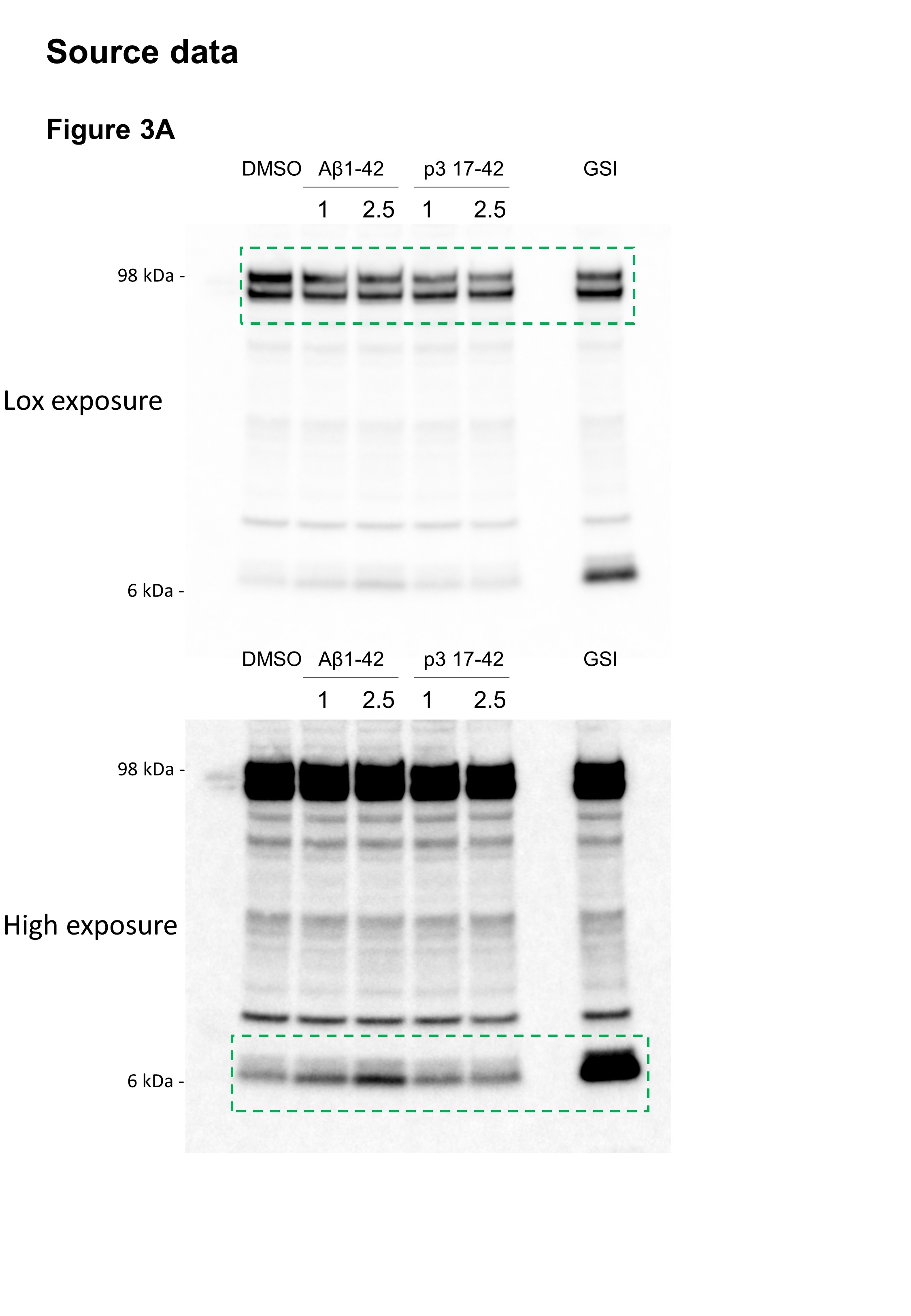

Supplement: Figure 3—source data 1. [file elife-90690-fig3-data1.zip › Figure 3 Source Data 1_panel A.JPG]

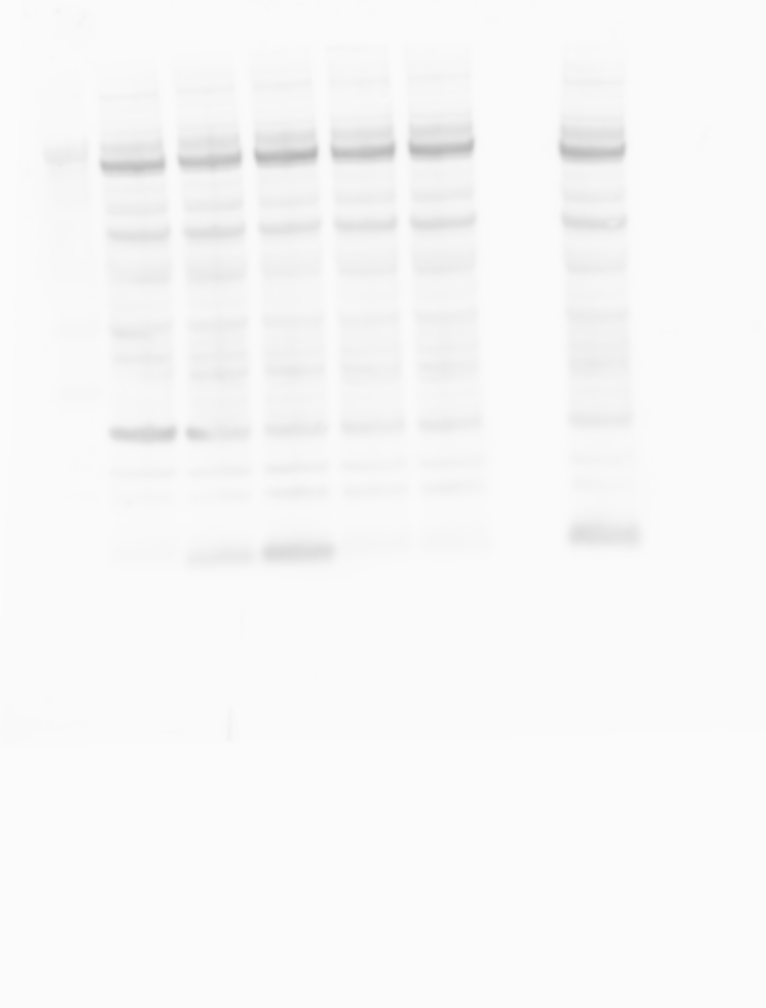

Supplement: Figure 3—source data 1. [file elife-90690-fig3-data1.zip › Figure 3 Source Data 2_org.gel]

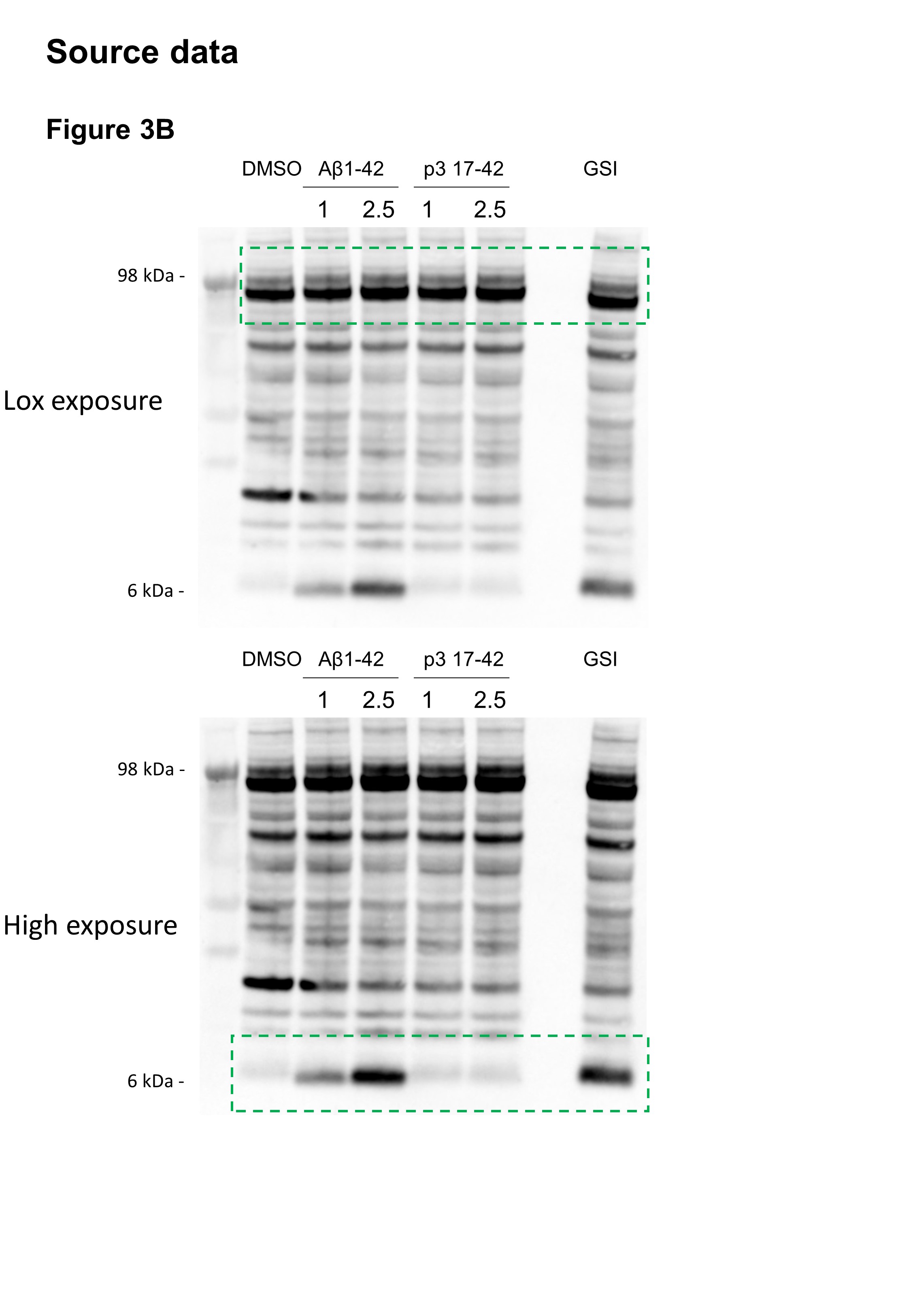

Supplement: Figure 3—source data 1. [file elife-90690-fig3-data1.zip › Figure 3 Source Data 2_panel B.JPG]

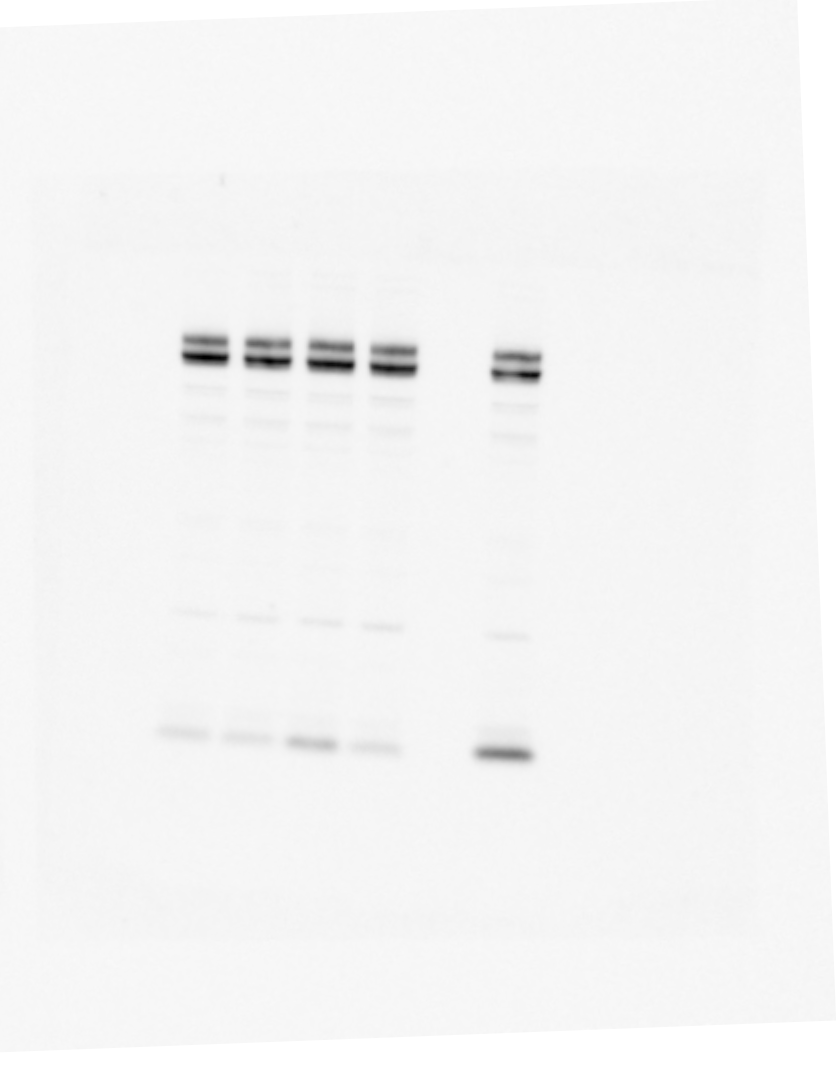

Supplement: Figure 3—source data 1. [file elife-90690-fig3-data1.zip › Figure 3 Source Data 3_org.gel]

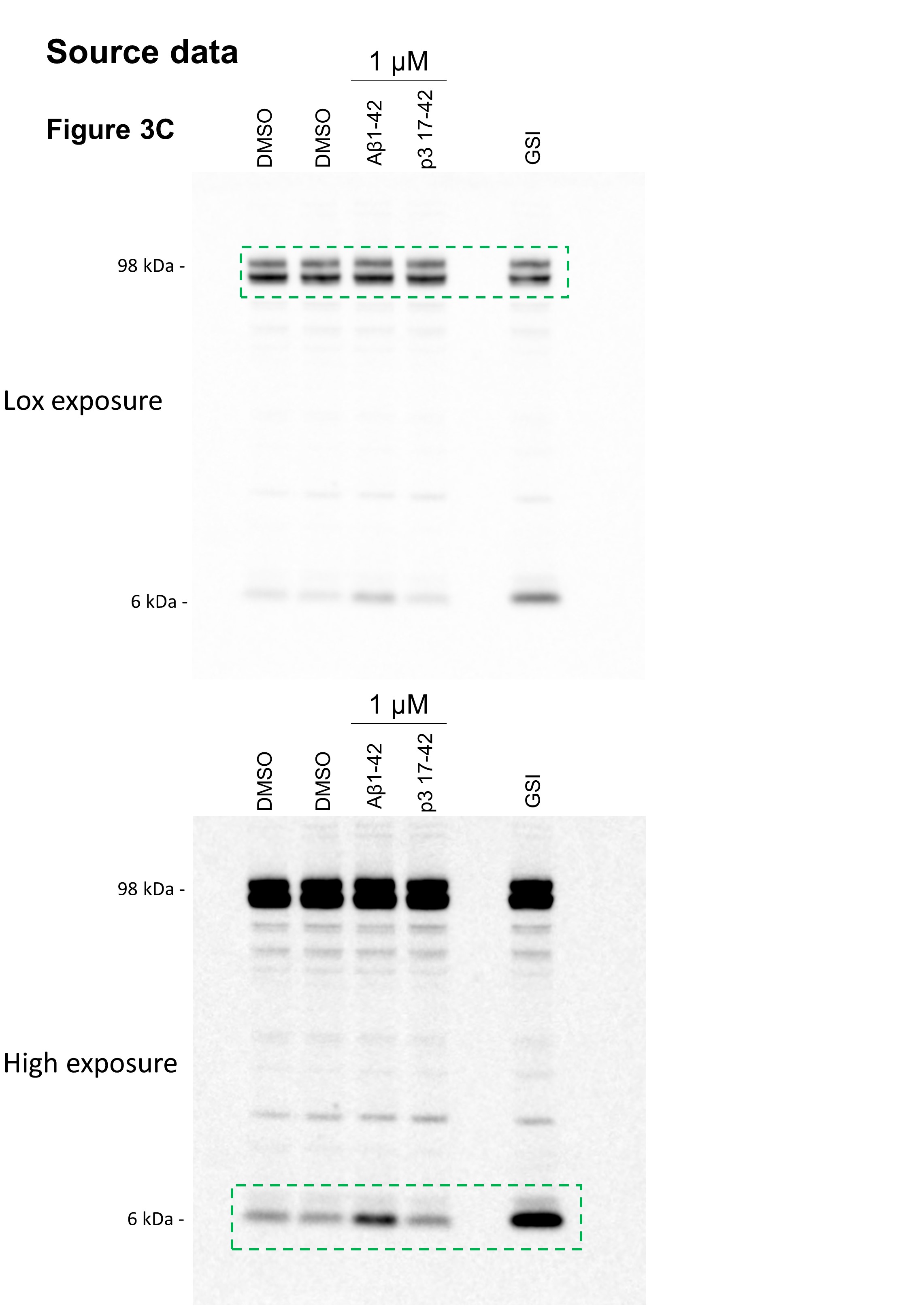

Supplement: Figure 3—source data 1. [file elife-90690-fig3-data1.zip › Figure 3 Source Data 3_panel C.JPG]

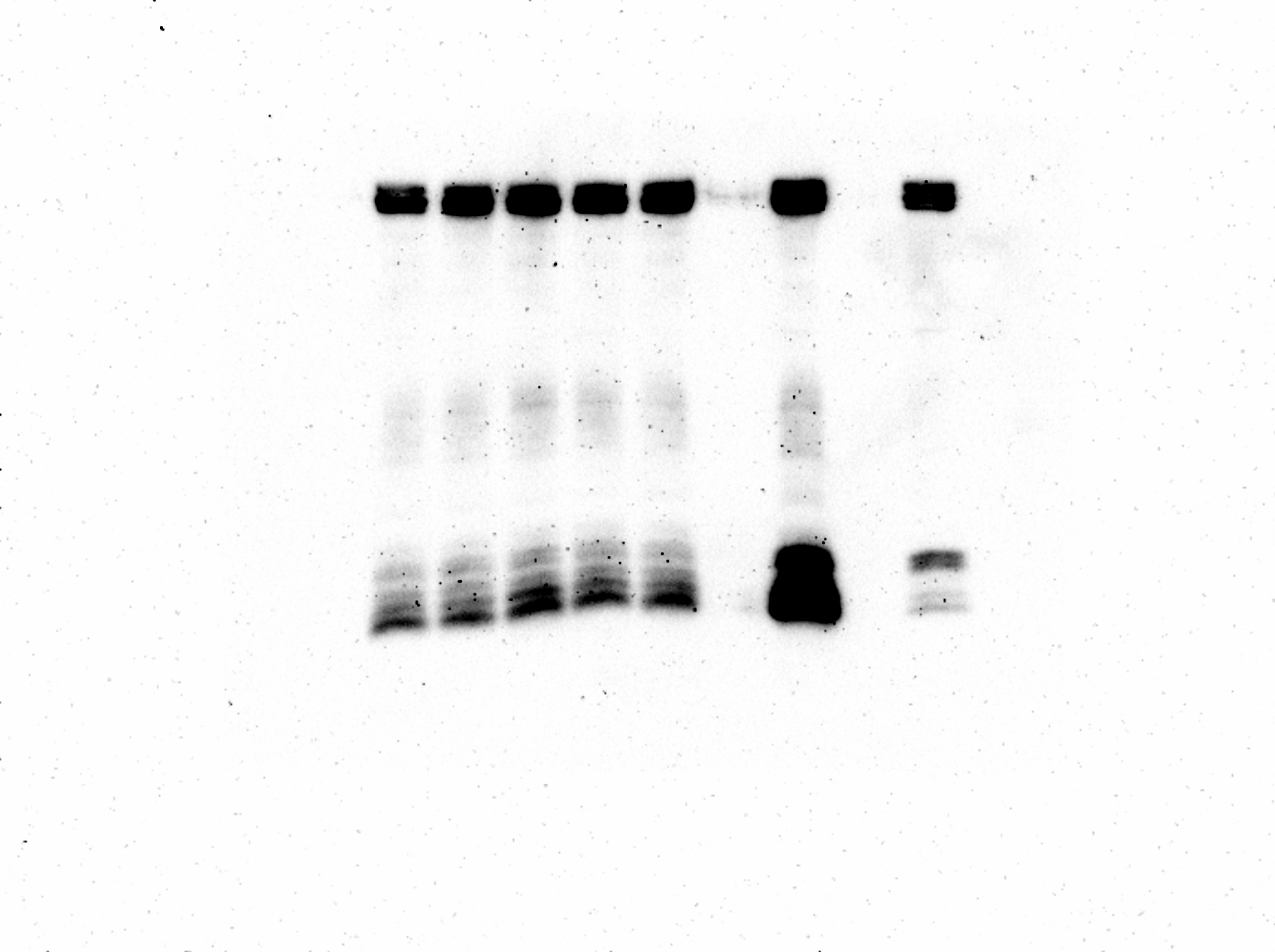

Supplement: Figure 3—source data 1. [file elife-90690-fig3-data1.zip › Figure 3 Source Data 4_org1.tif]

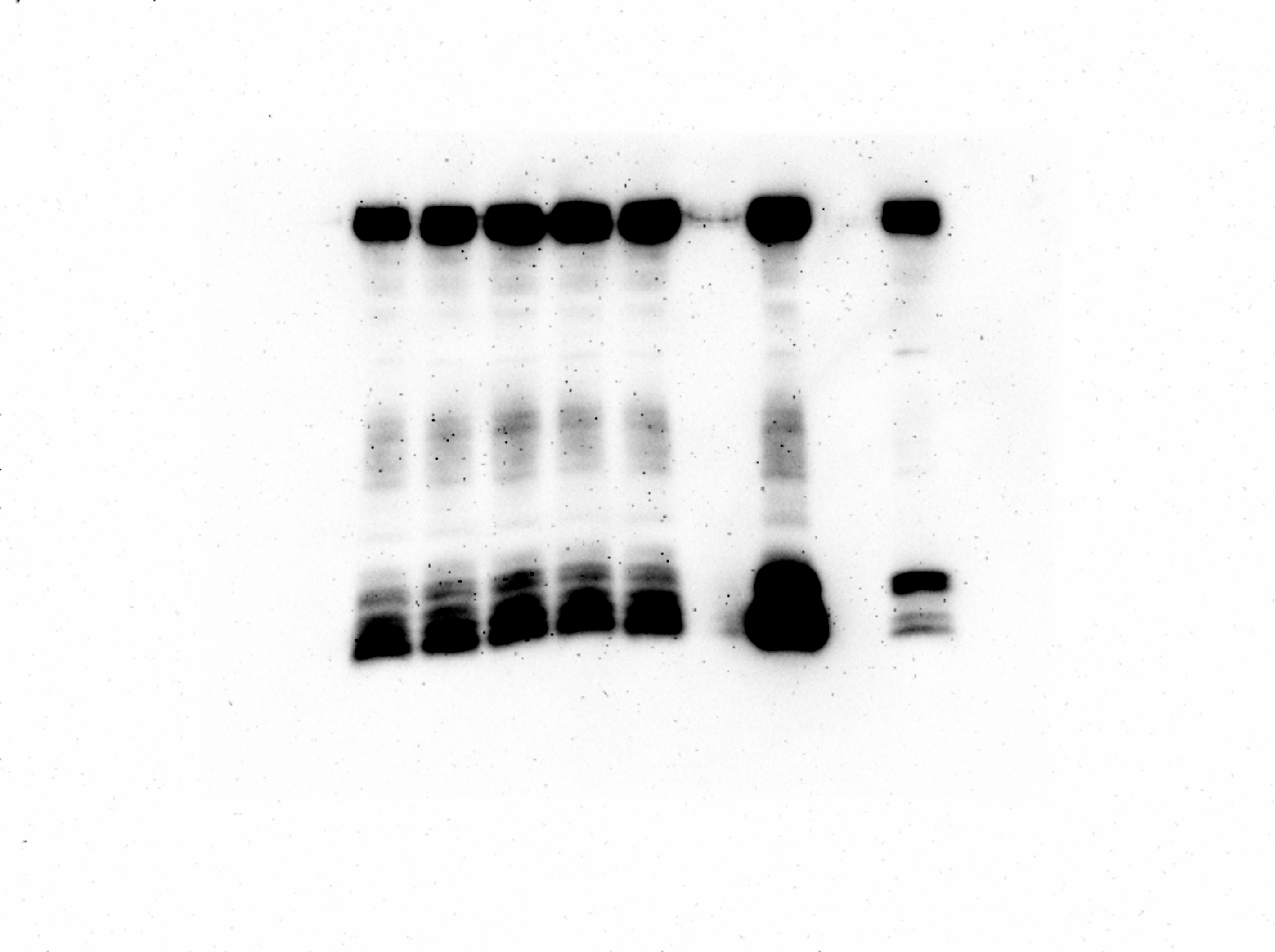

Supplement: Figure 3—source data 1. [file elife-90690-fig3-data1.zip › Figure 3 Source Data 4_org2.tif]

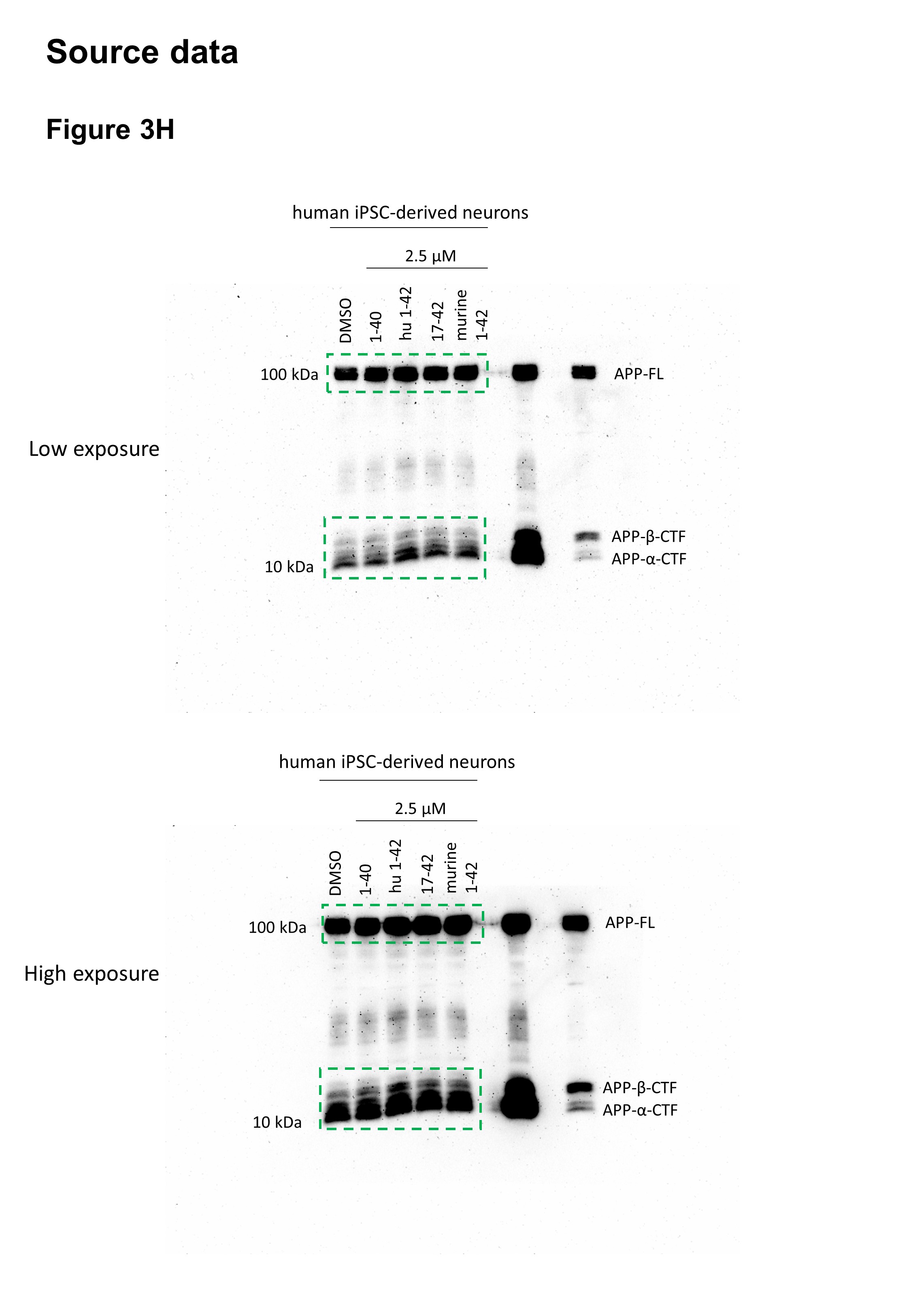

Supplement: Figure 3—source data 1. [file elife-90690-fig3-data1.zip › Figure 3 Source Data 4_panel H.JPG]

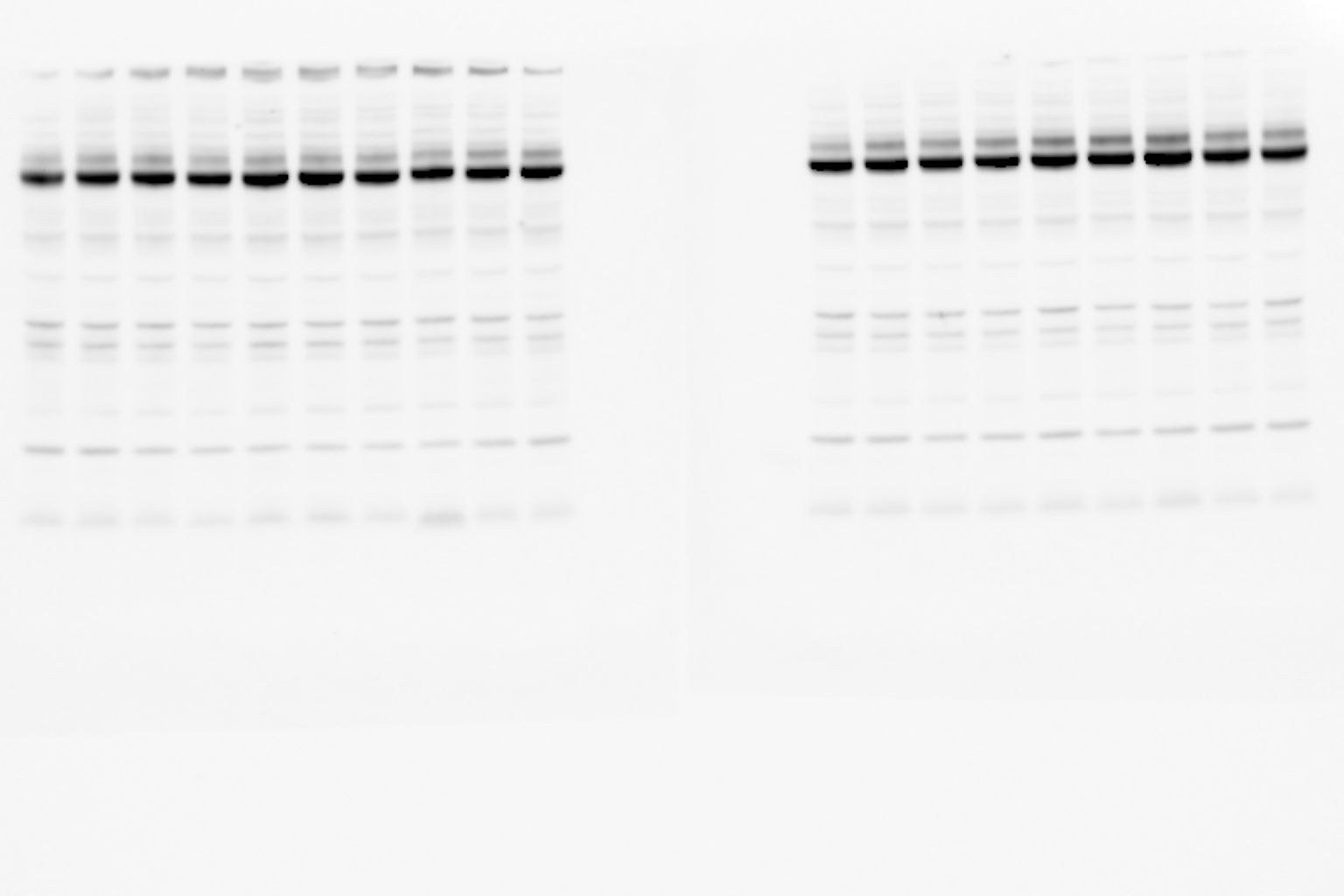

Supplement: Figure 4—source data 1. [file elife-90690-fig4-data1.zip › Figure 4 Source Data 1_org.gel]

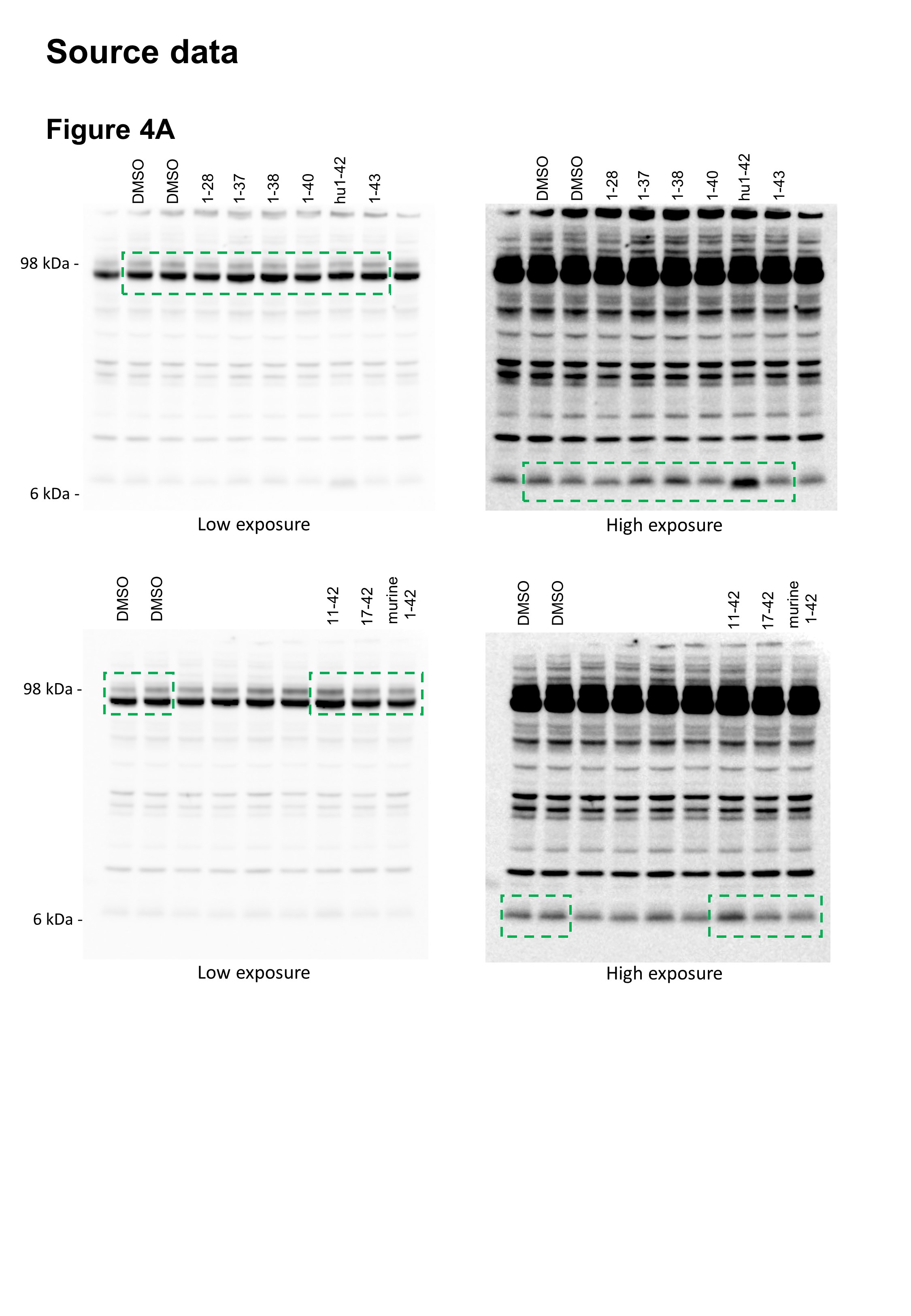

Supplement: Figure 4—source data 1. [file elife-90690-fig4-data1.zip › Figure 4 Source Data 1_panel A.JPG]

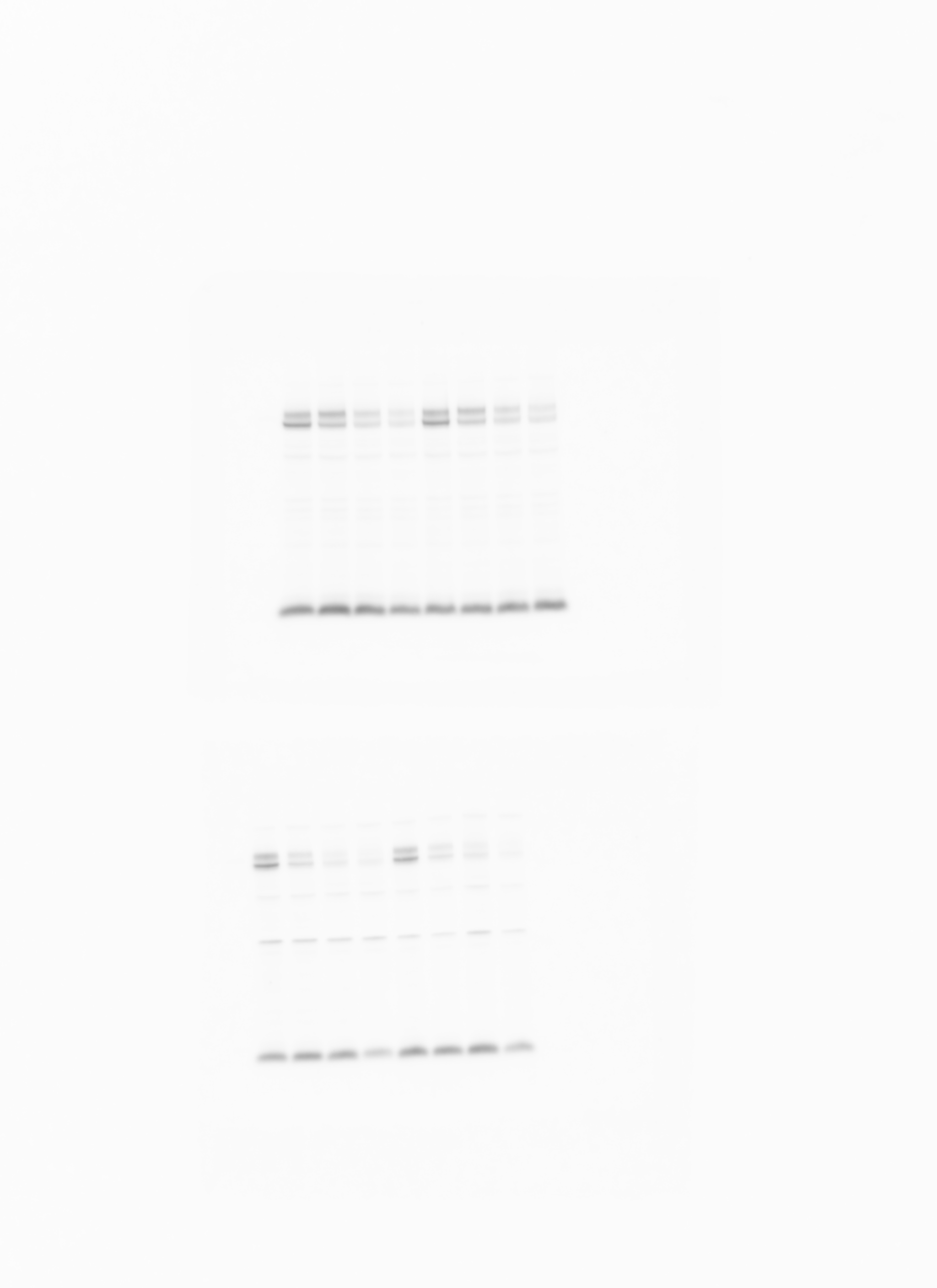

Supplement: Figure 6—source data 1. [file elife-90690-fig6-data1.zip › Figure 6 Source Data 1_org.tif]

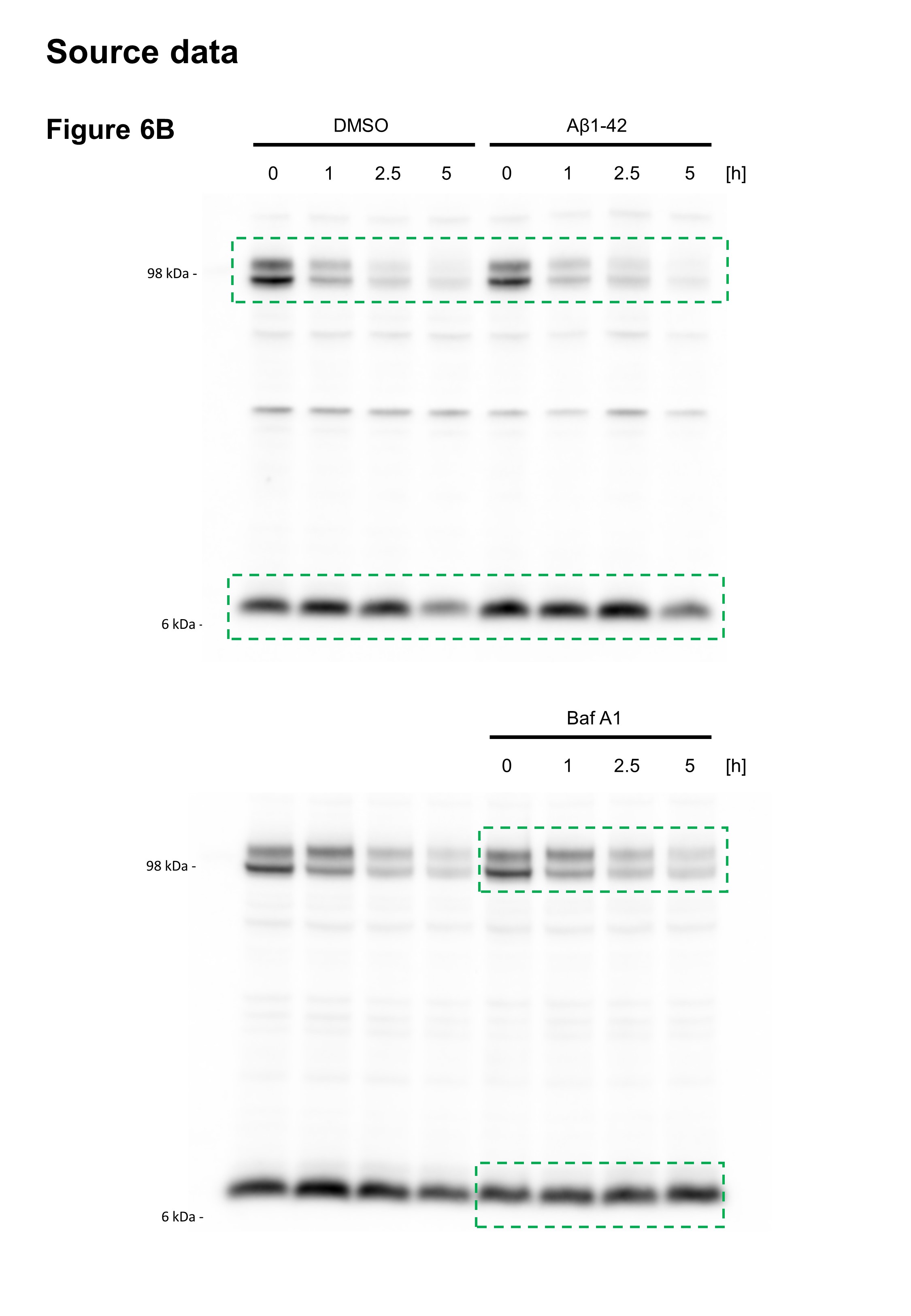

Supplement: Figure 6—source data 1. [file elife-90690-fig6-data1.zip › Figure 6 Source Data 1_panel B.JPG]

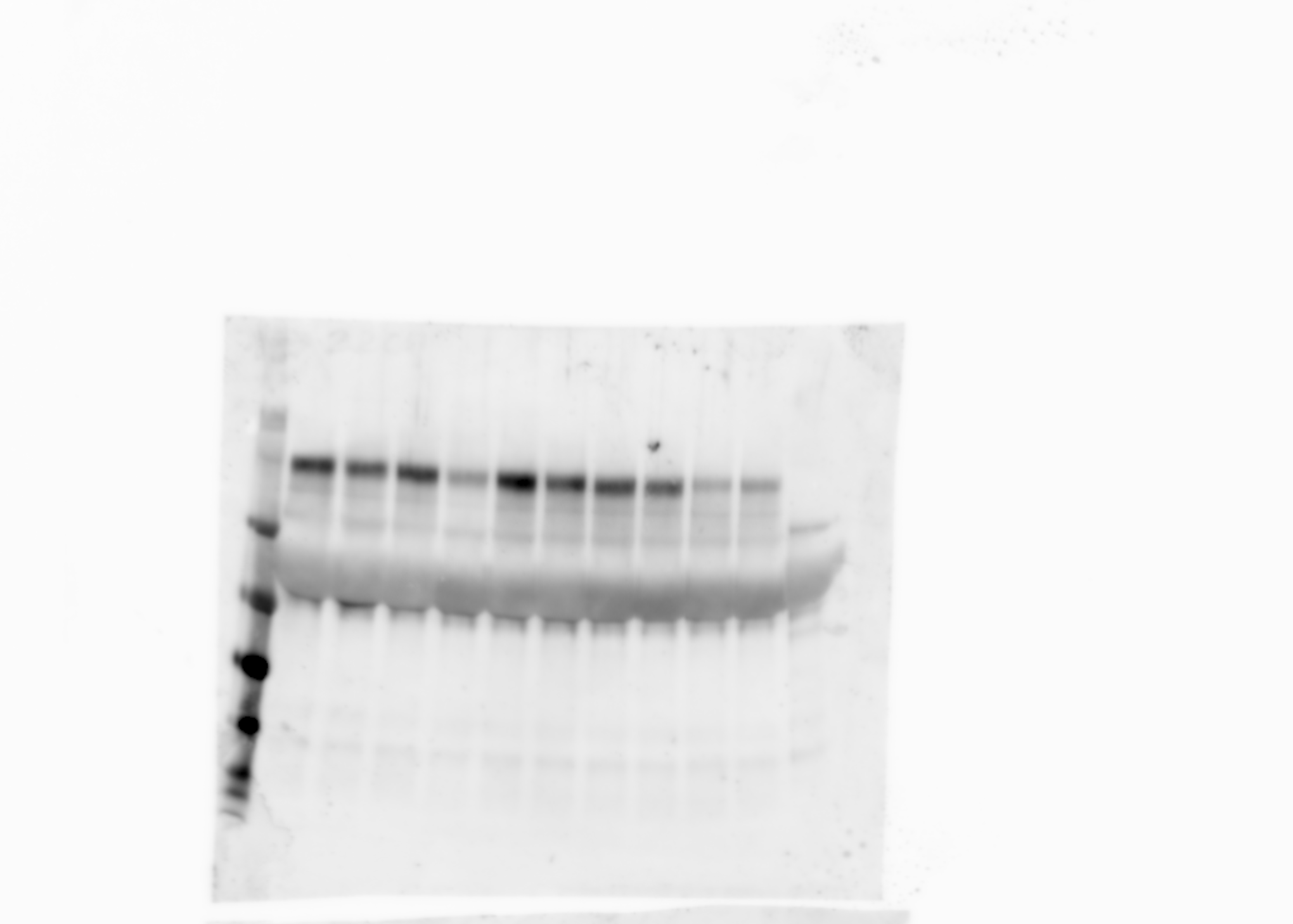

Supplement: Figure 6—figure supplement 1—source data 1. [file elife-90690-fig6-figsupp1-data1.zip › Figure 6 Source Data 2_org.tif]

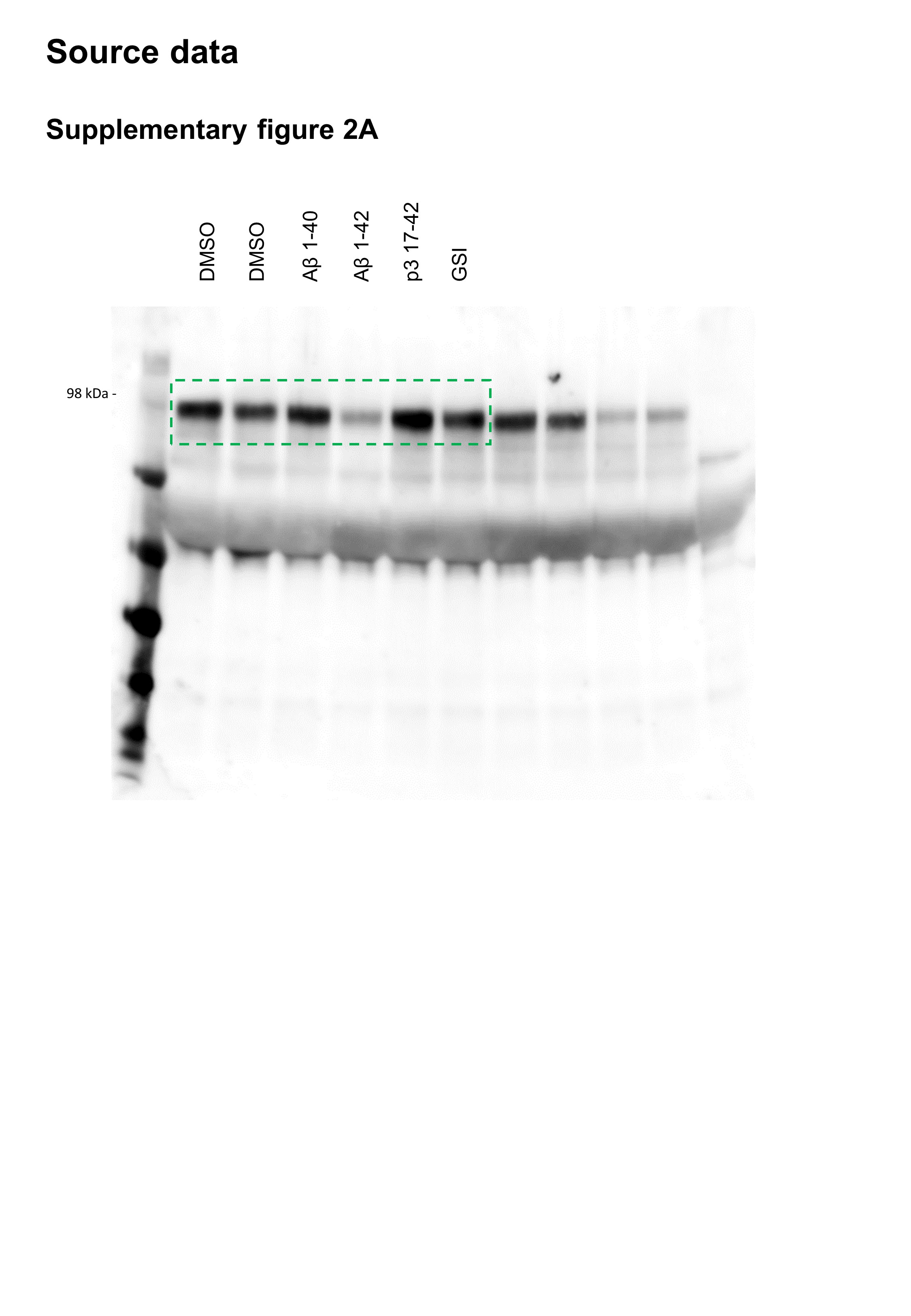

Supplement: Figure 6—figure supplement 1—source data 1. [file elife-90690-fig6-figsupp1-data1.zip › Figure 6 Source Data 2_panel A.JPG]

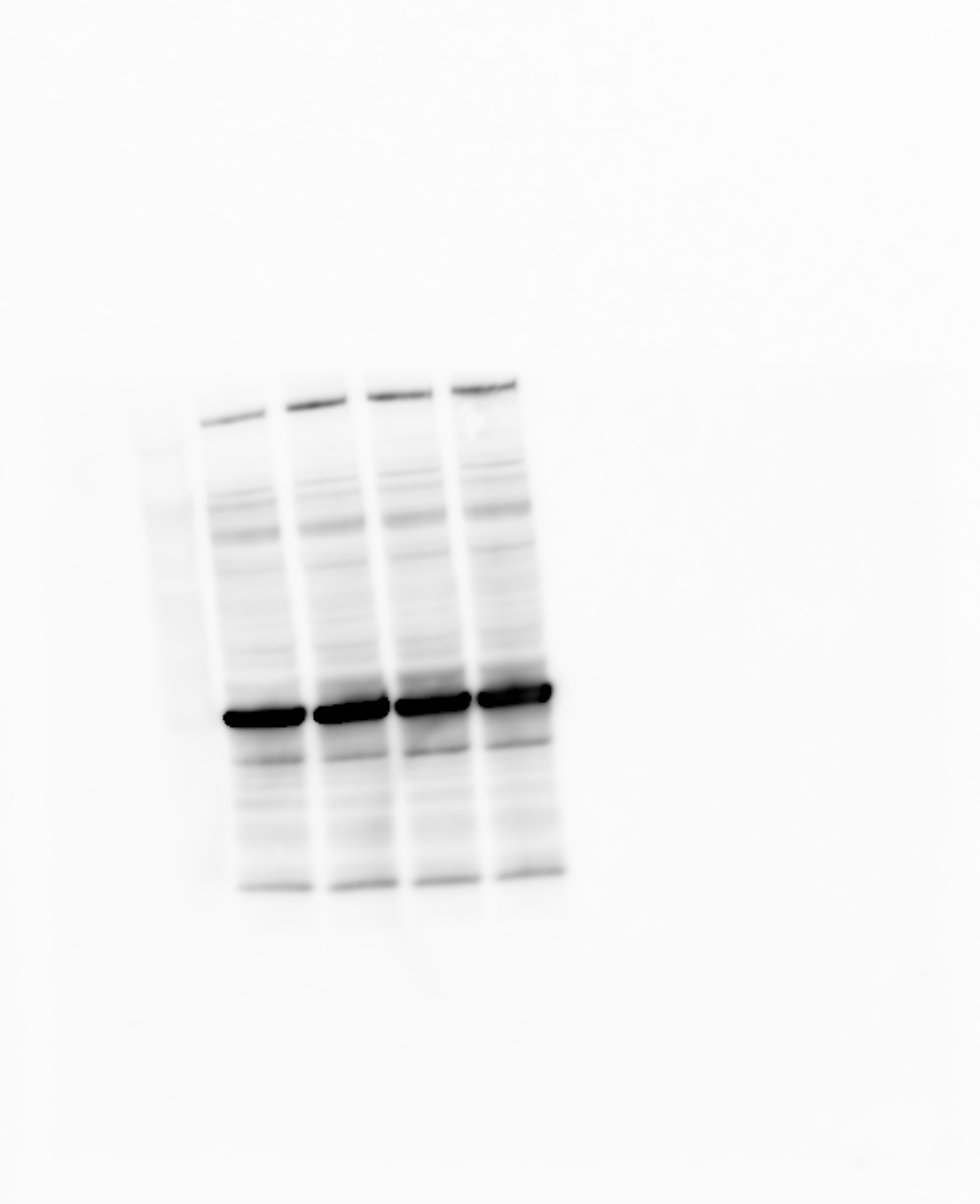

Supplement: Figure 6—figure supplement 1—source data 1. [file elife-90690-fig6-figsupp1-data1.zip › Figure 6 Source Data 3_org 1.tif]

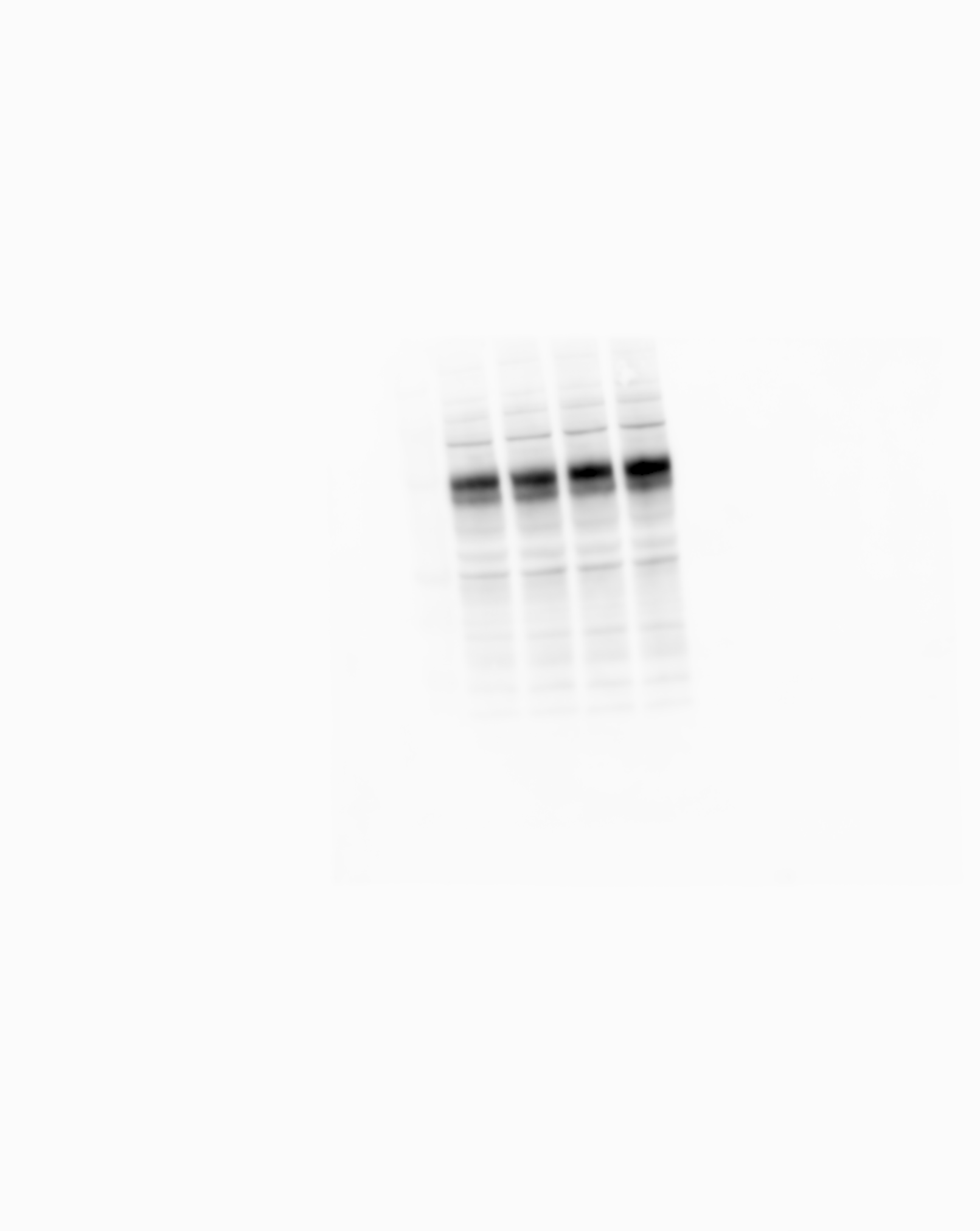

Supplement: Figure 6—figure supplement 1—source data 1. [file elife-90690-fig6-figsupp1-data1.zip › Figure 6 Source Data 3_org 2.tif]

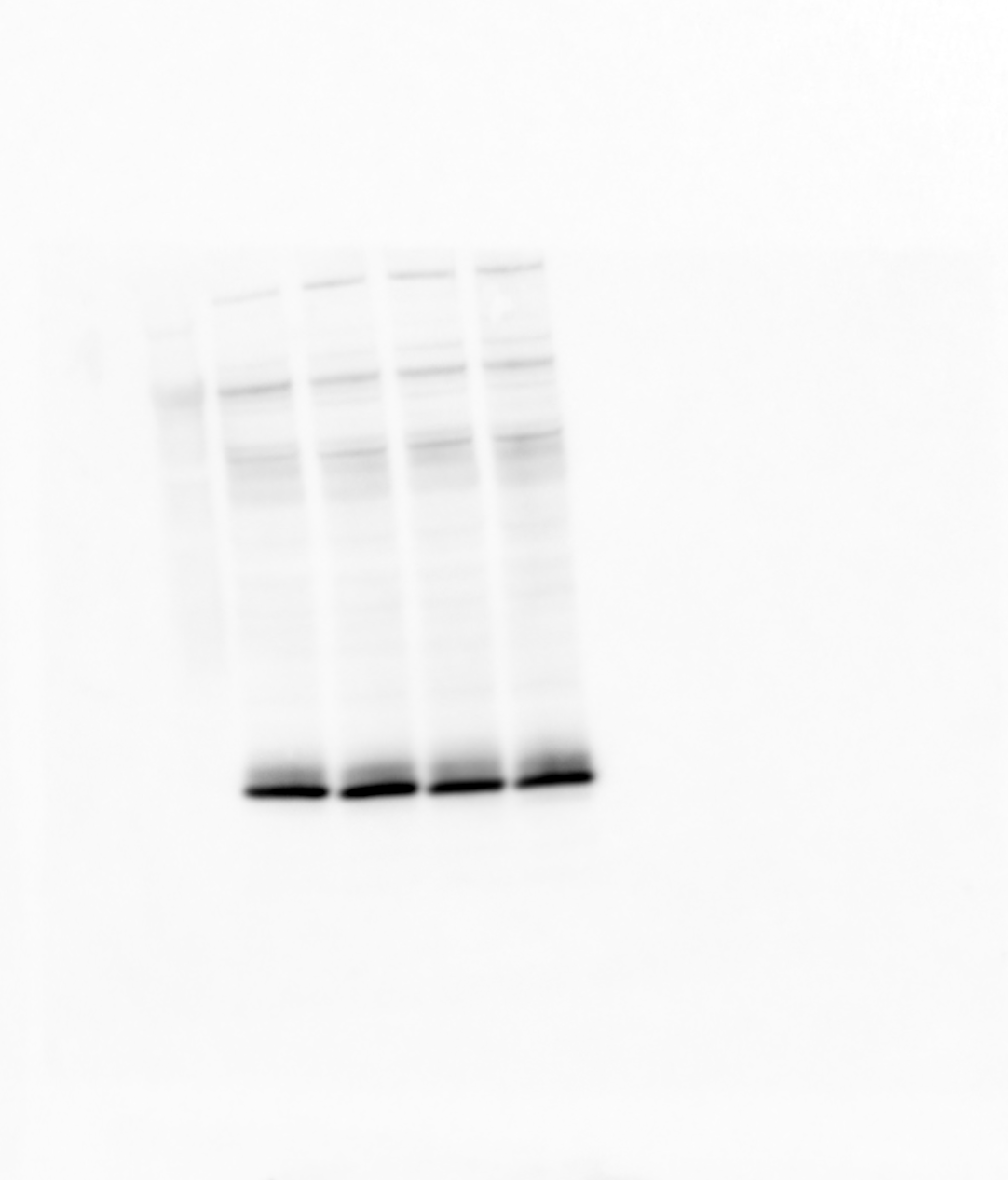

Supplement: Figure 6—figure supplement 1—source data 1. [file elife-90690-fig6-figsupp1-data1.zip › Figure 6 Source Data 3_org 3.tif]

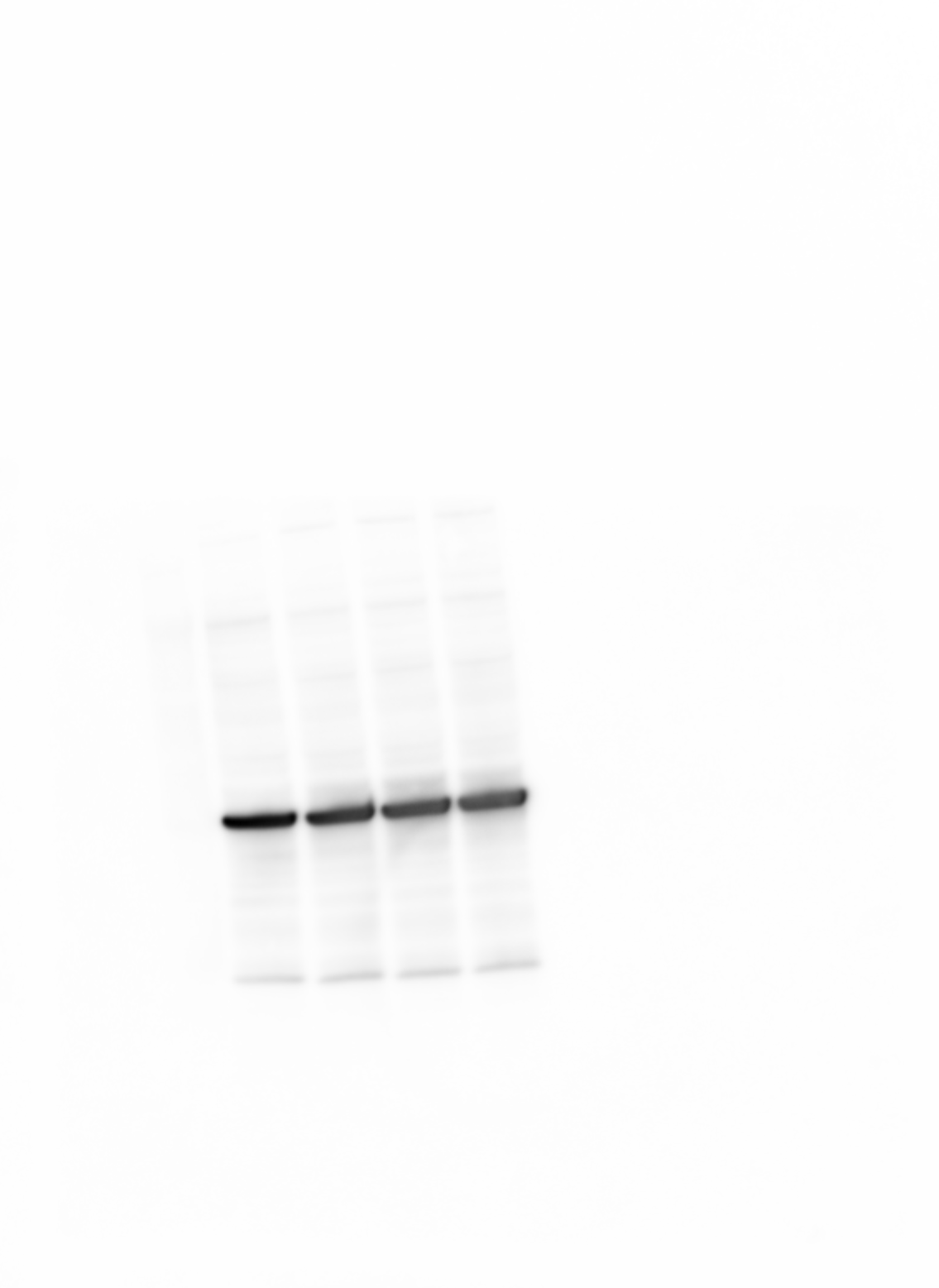

Supplement: Figure 6—figure supplement 1—source data 1. [file elife-90690-fig6-figsupp1-data1.zip › Figure 6 Source Data 3_org 4.tif]

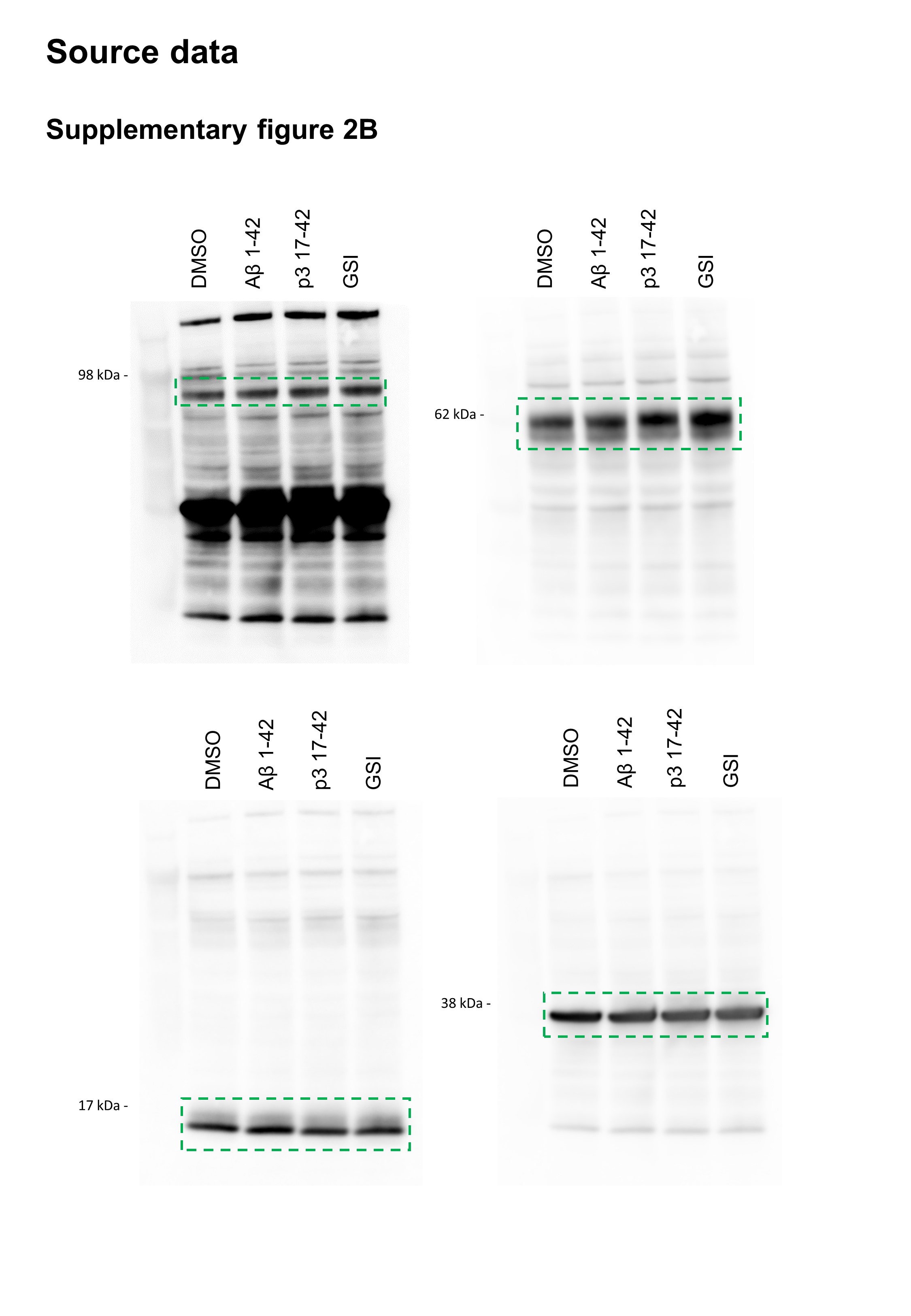

Supplement: Figure 6—figure supplement 1—source data 1. [file elife-90690-fig6-figsupp1-data1.zip › Figure 6 Source Data 3_panel B.JPG]

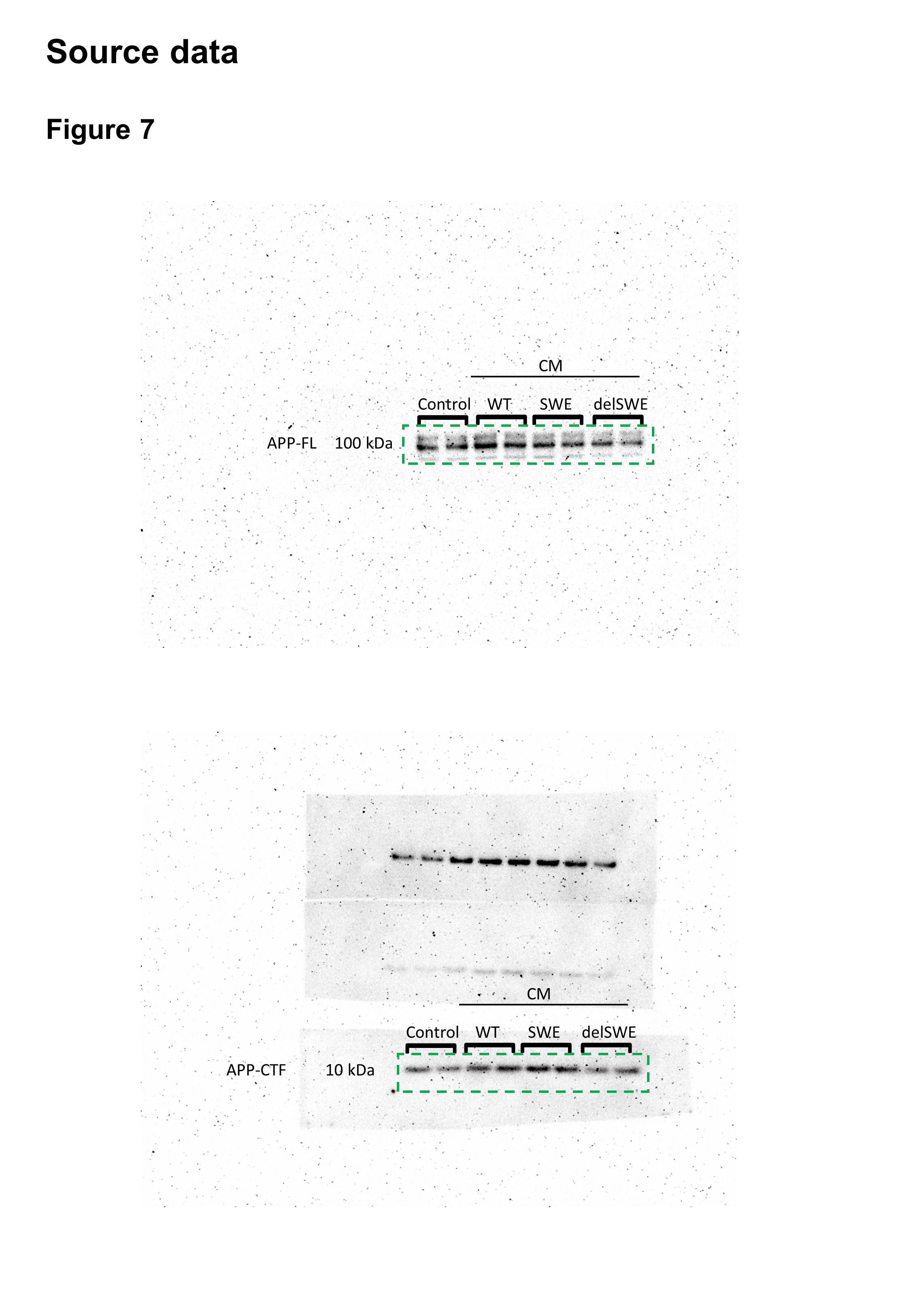

Supplement: Figure 7—source data 1. [file elife-90690-fig7-data1.zip › Figure 7 Source Data 1.JPG]

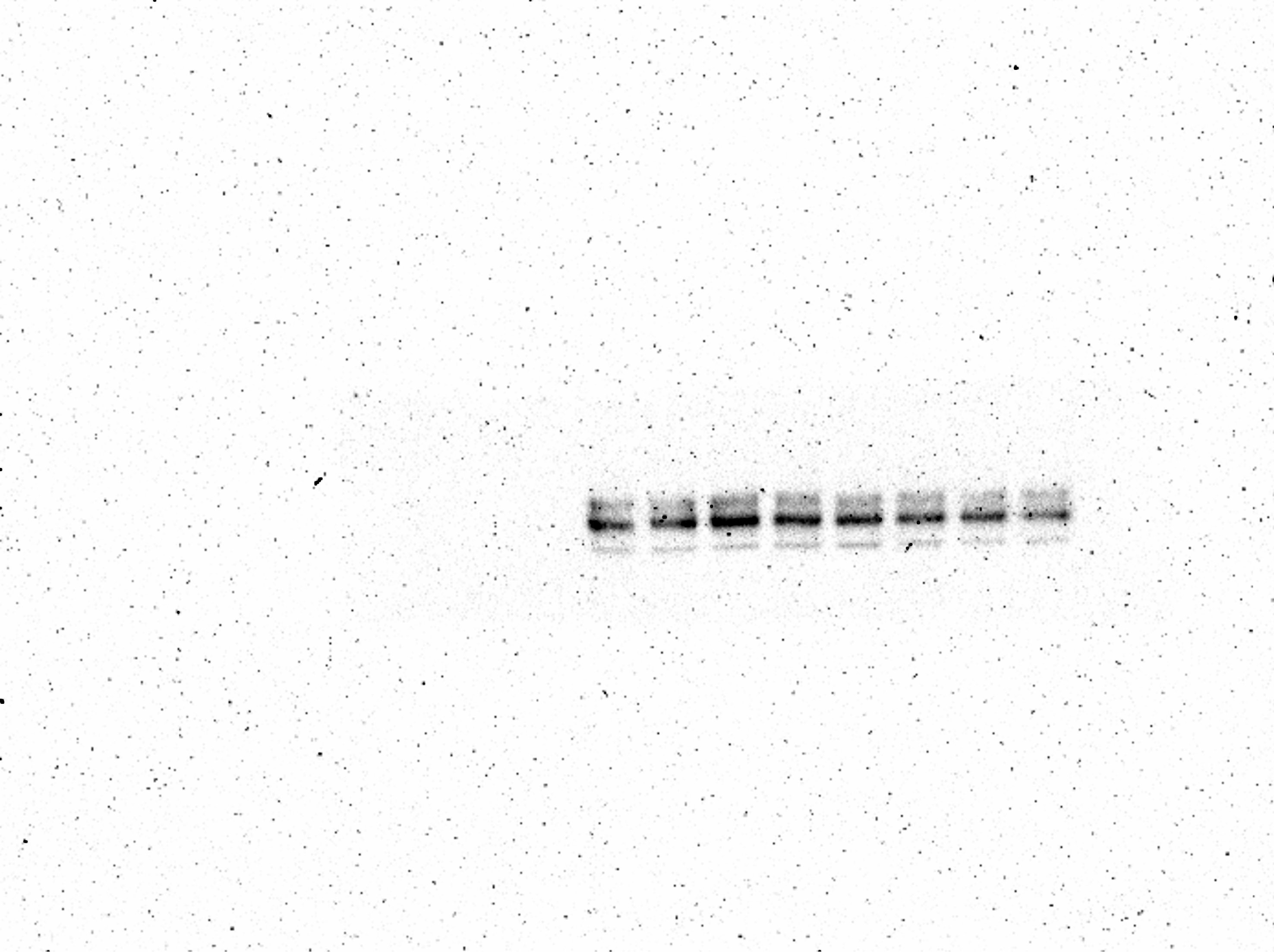

Supplement: Figure 7—source data 1. [file elife-90690-fig7-data1.zip › Figure 7 Source Data 1_org1.tif]

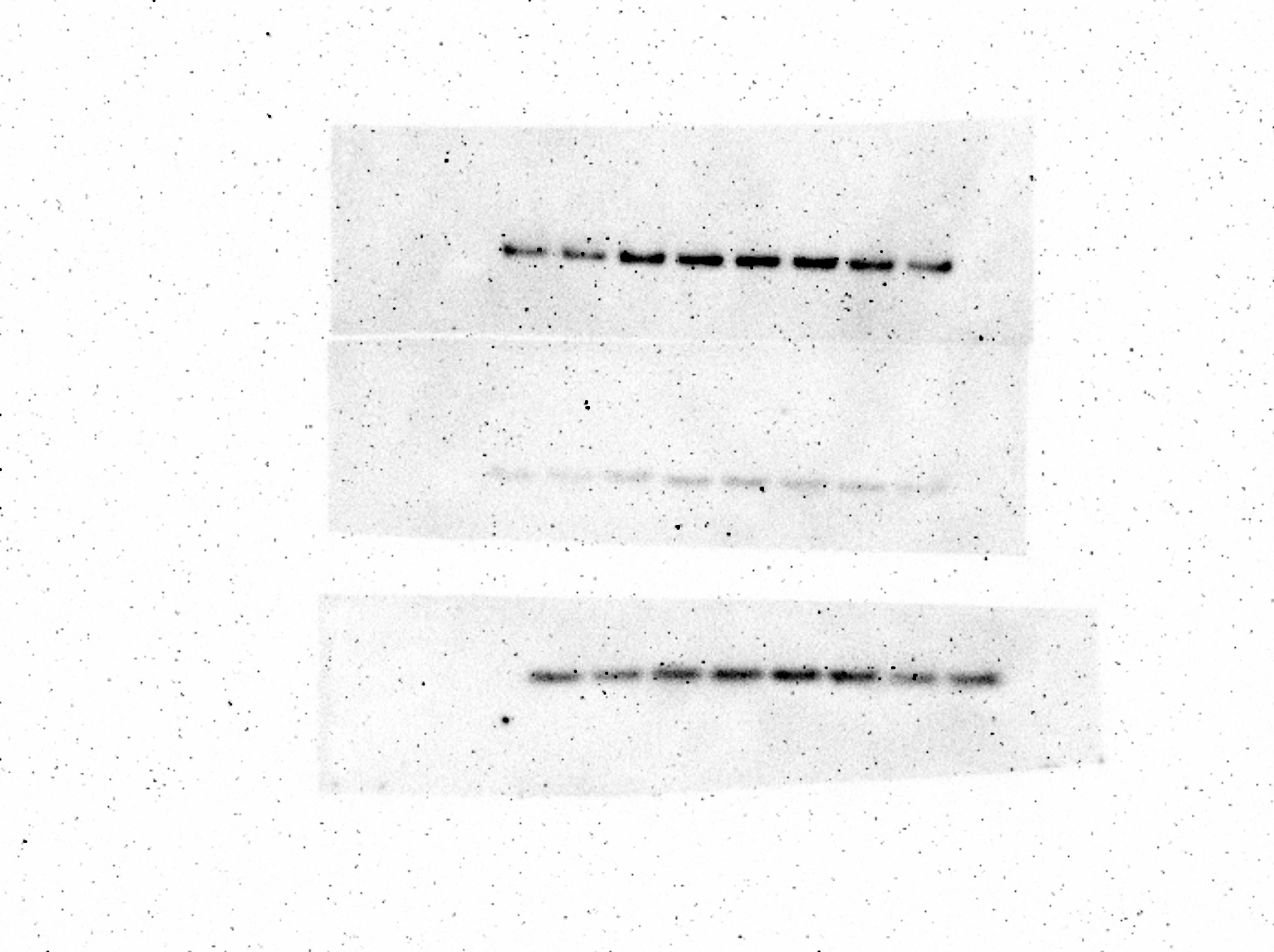

Supplement: Figure 7—source data 1. [file elife-90690-fig7-data1.zip › Figure 7 Source Data 1_org2.tif]

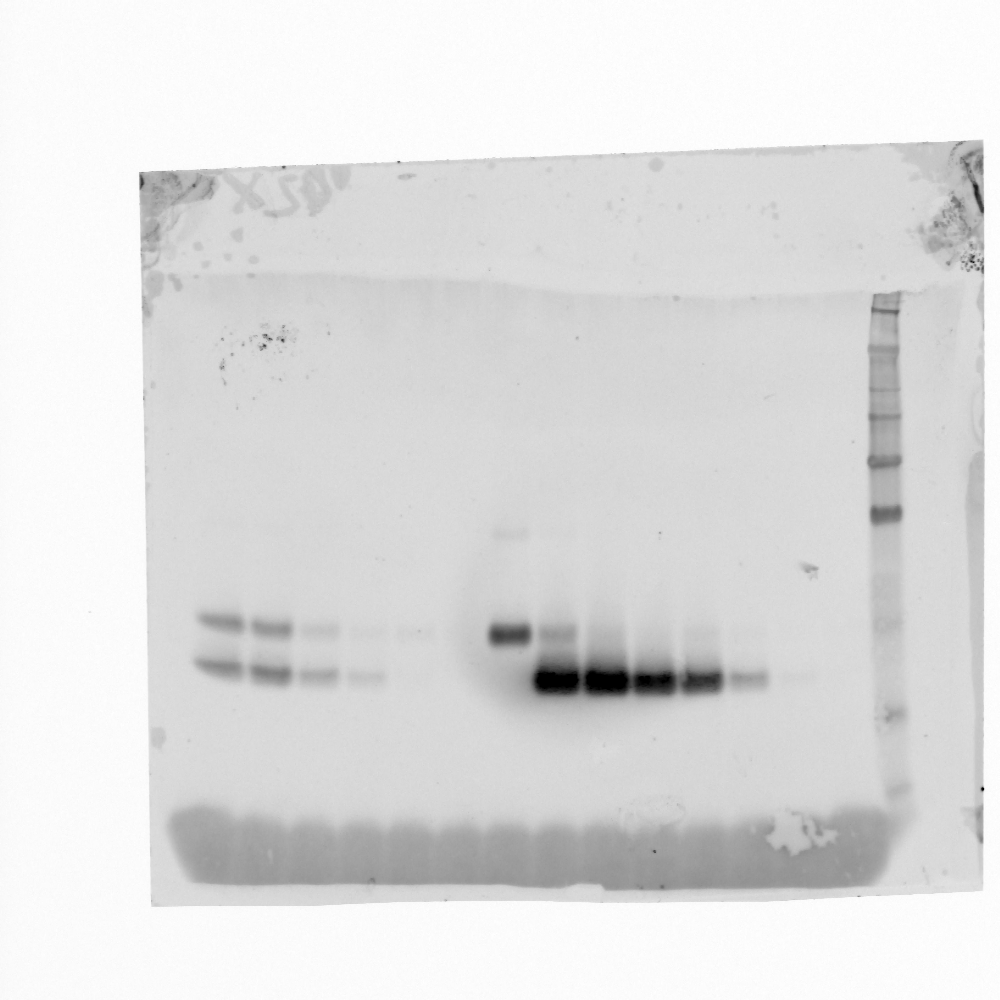

Supplement: Figure 8—source data 1. [file elife-90690-fig8-data1.zip › Figure 8 Source Data 1_org 1.gel]

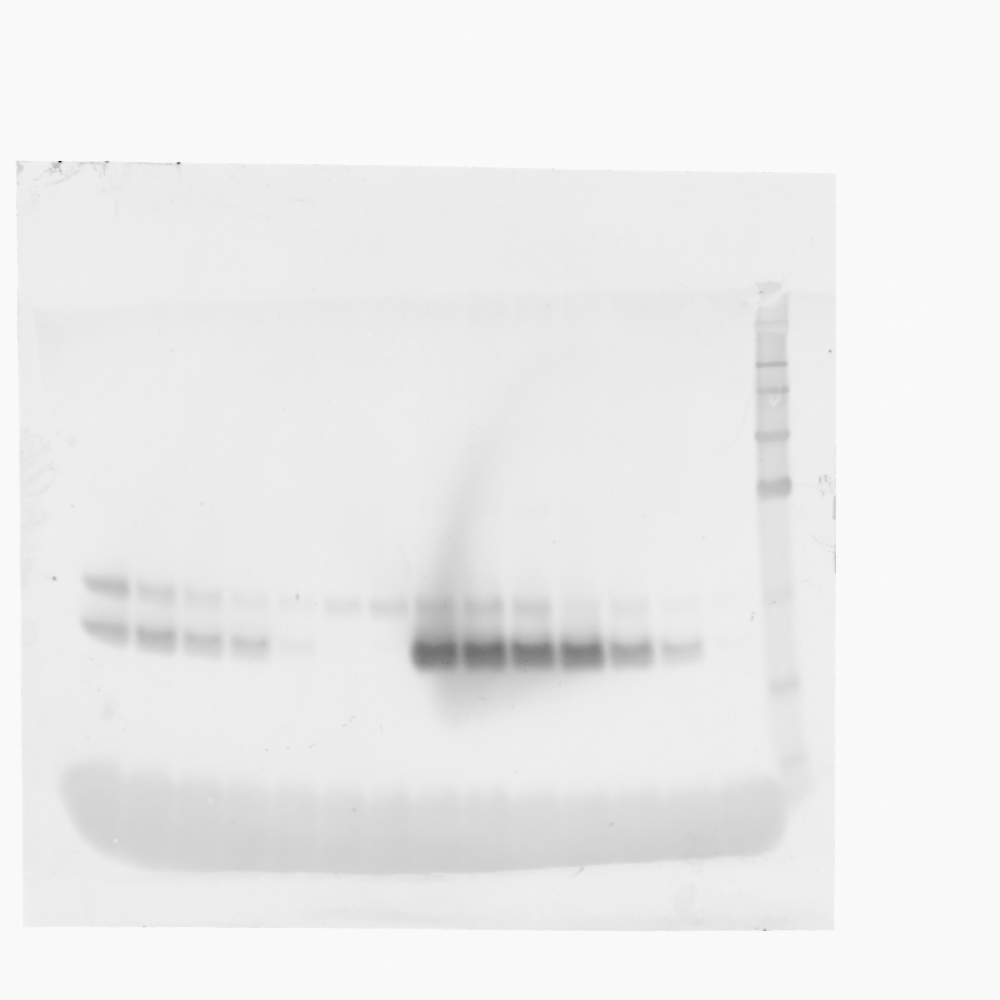

Supplement: Figure 8—source data 1. [file elife-90690-fig8-data1.zip › Figure 8 Source Data 1_org 2.gel]

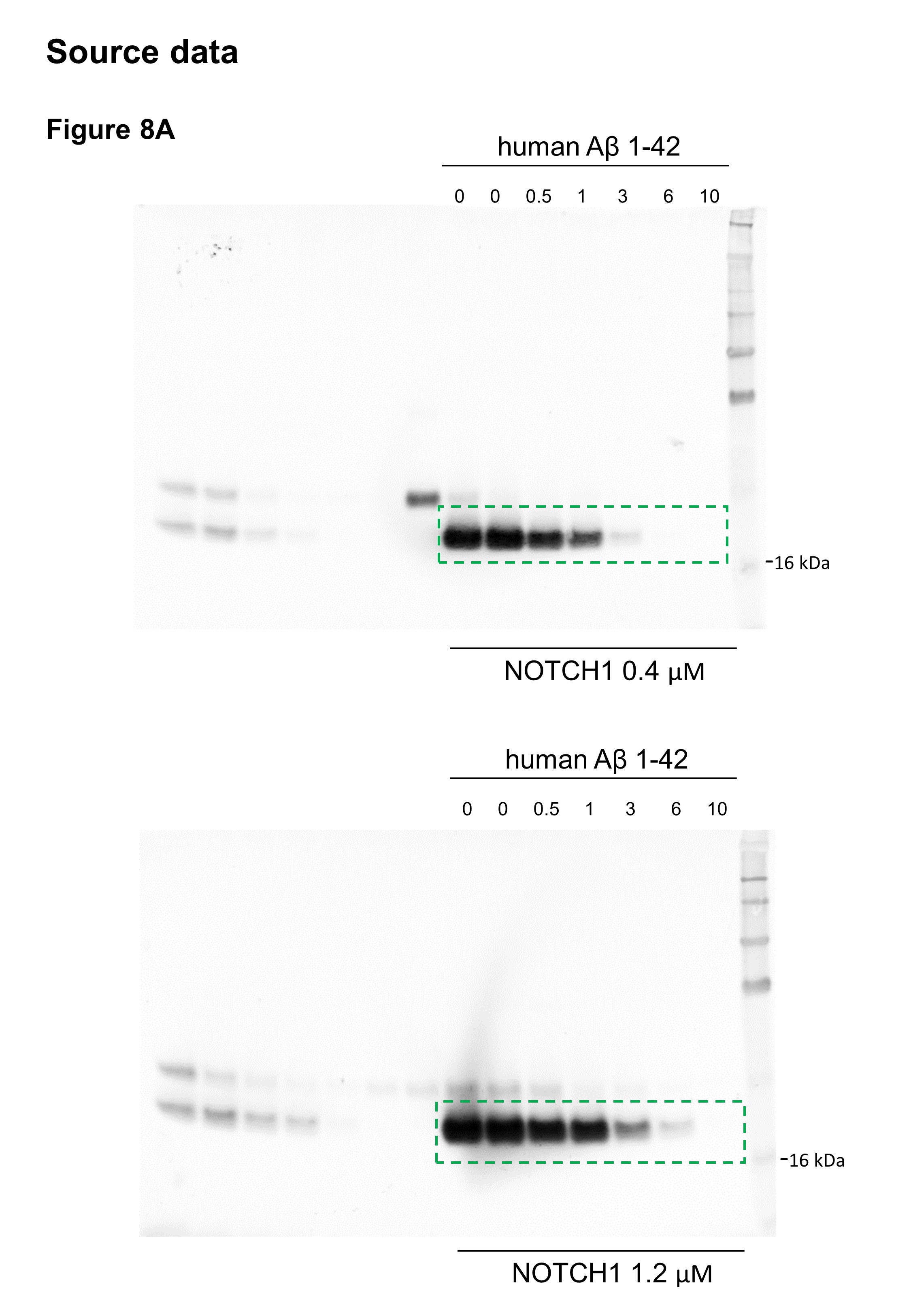

Supplement: Figure 8—source data 1. [file elife-90690-fig8-data1.zip › Figure 8 Source Data 1_panel A.JPG]

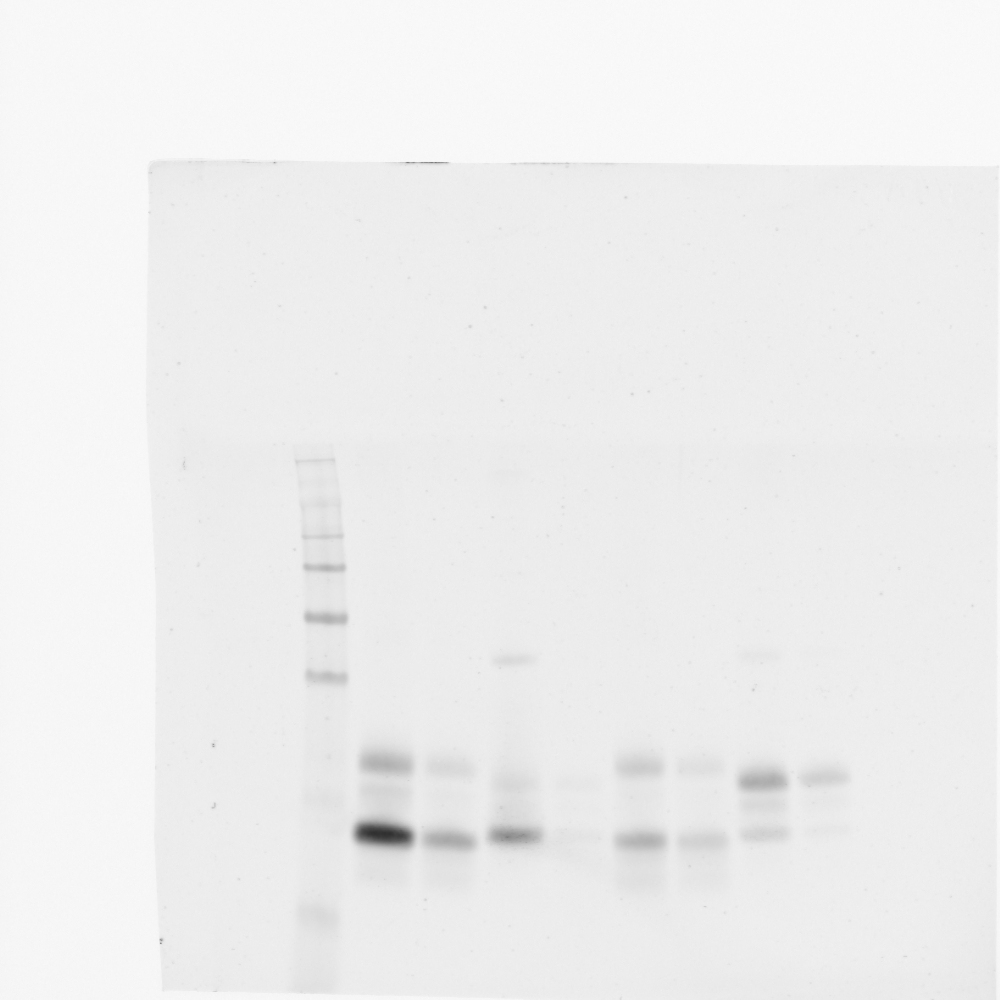

Supplement: Figure 8—source data 1. [file elife-90690-fig8-data1.zip › Figure 8 Source Data 2_org 1.gel]

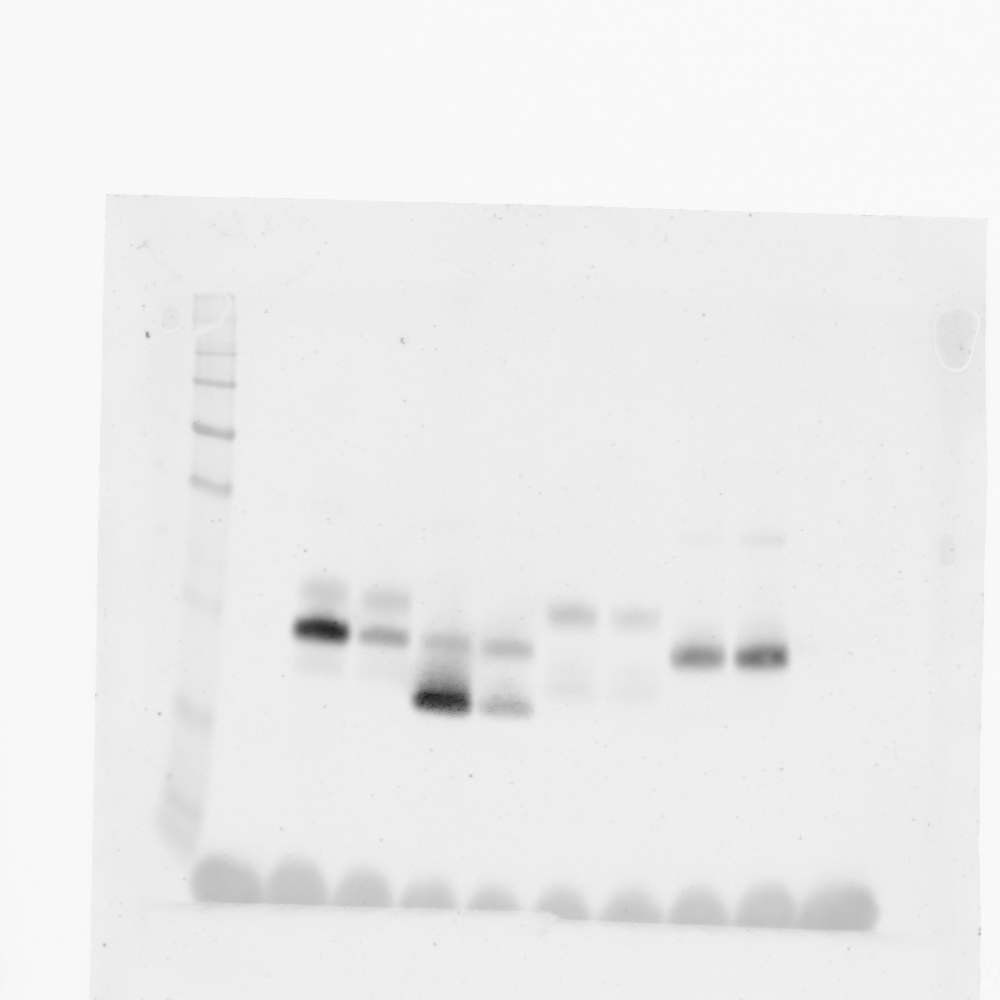

Supplement: Figure 8—source data 1. [file elife-90690-fig8-data1.zip › Figure 8 Source Data 2_org 2.gel]

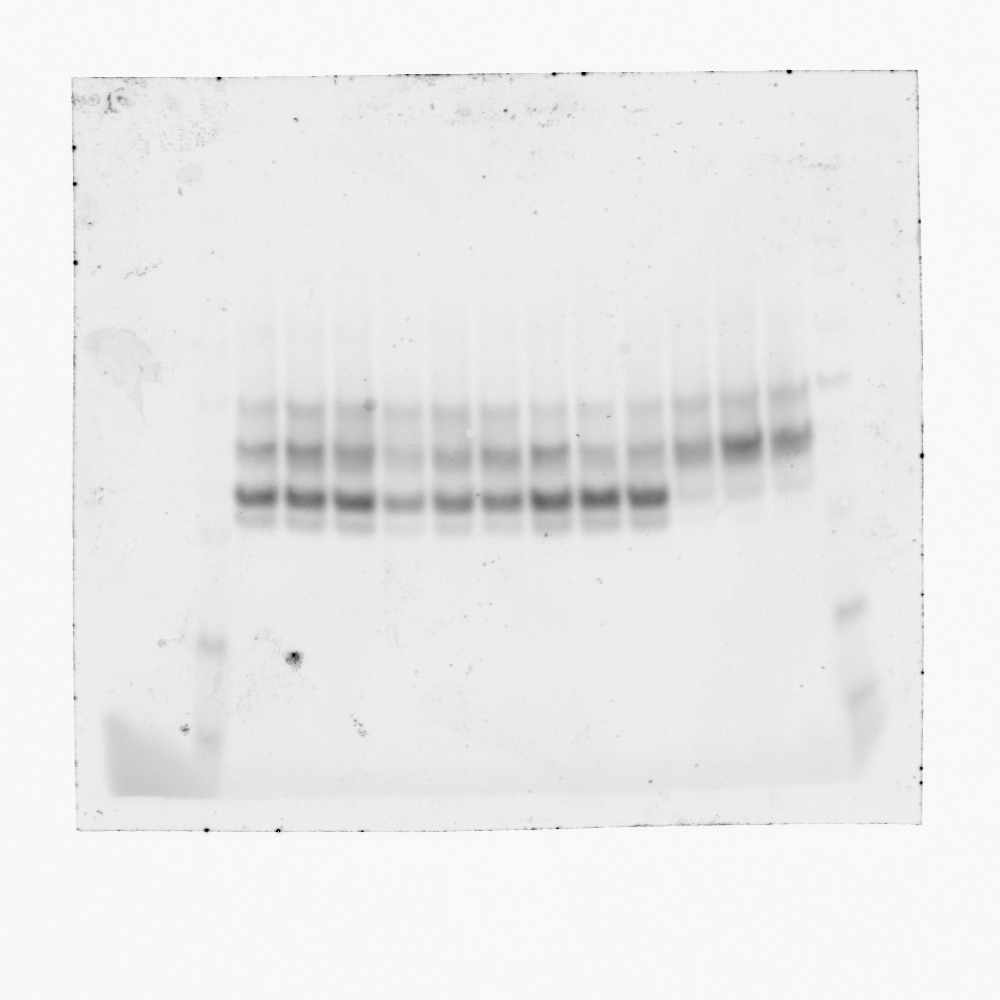

Supplement: Figure 8—source data 1. [file elife-90690-fig8-data1.zip › Figure 8 Source Data 2_org 3.gel]

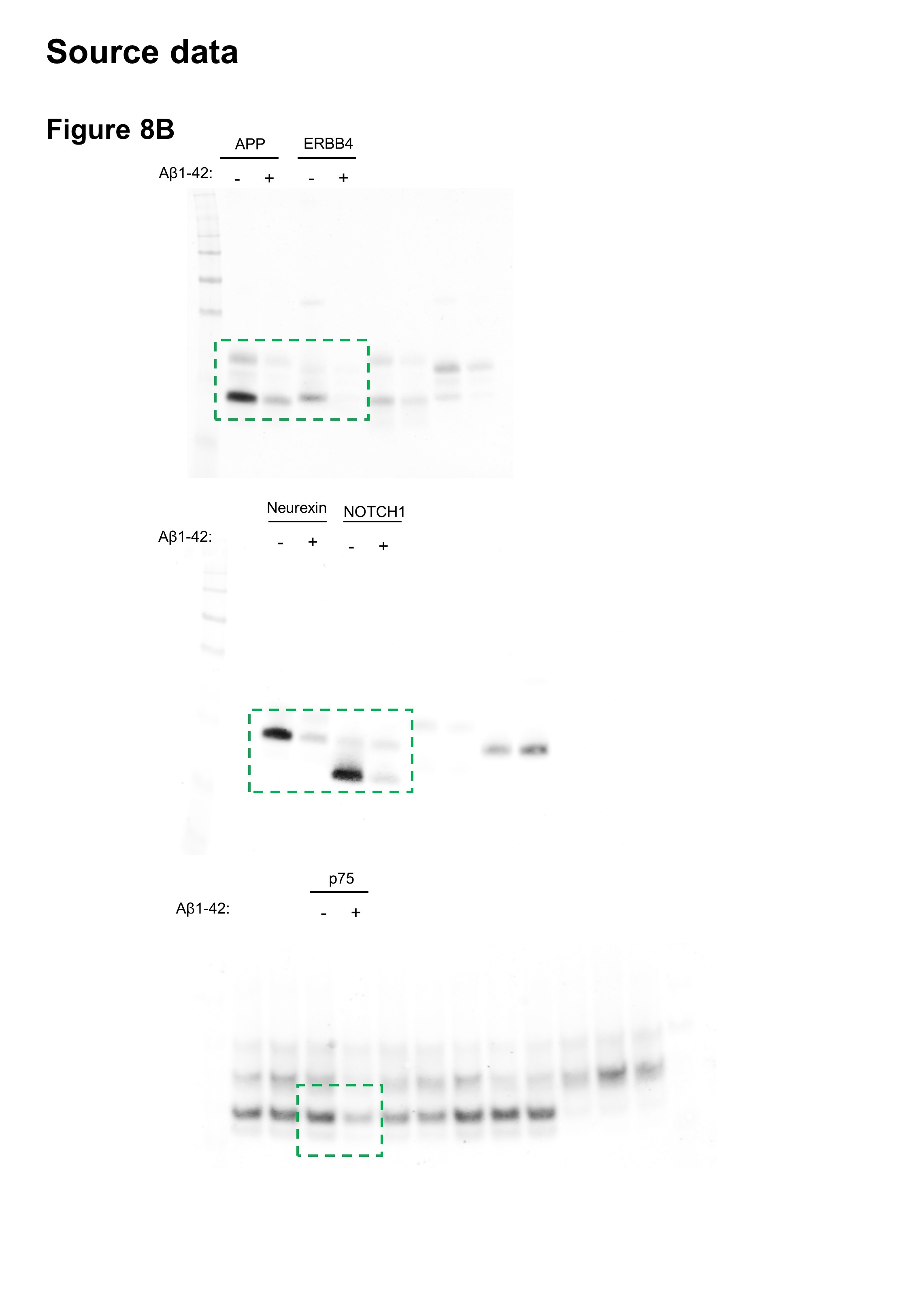

Supplement: Figure 8—source data 1. [file elife-90690-fig8-data1.zip › Figure 8 Source Data 2_panel B.jpg]

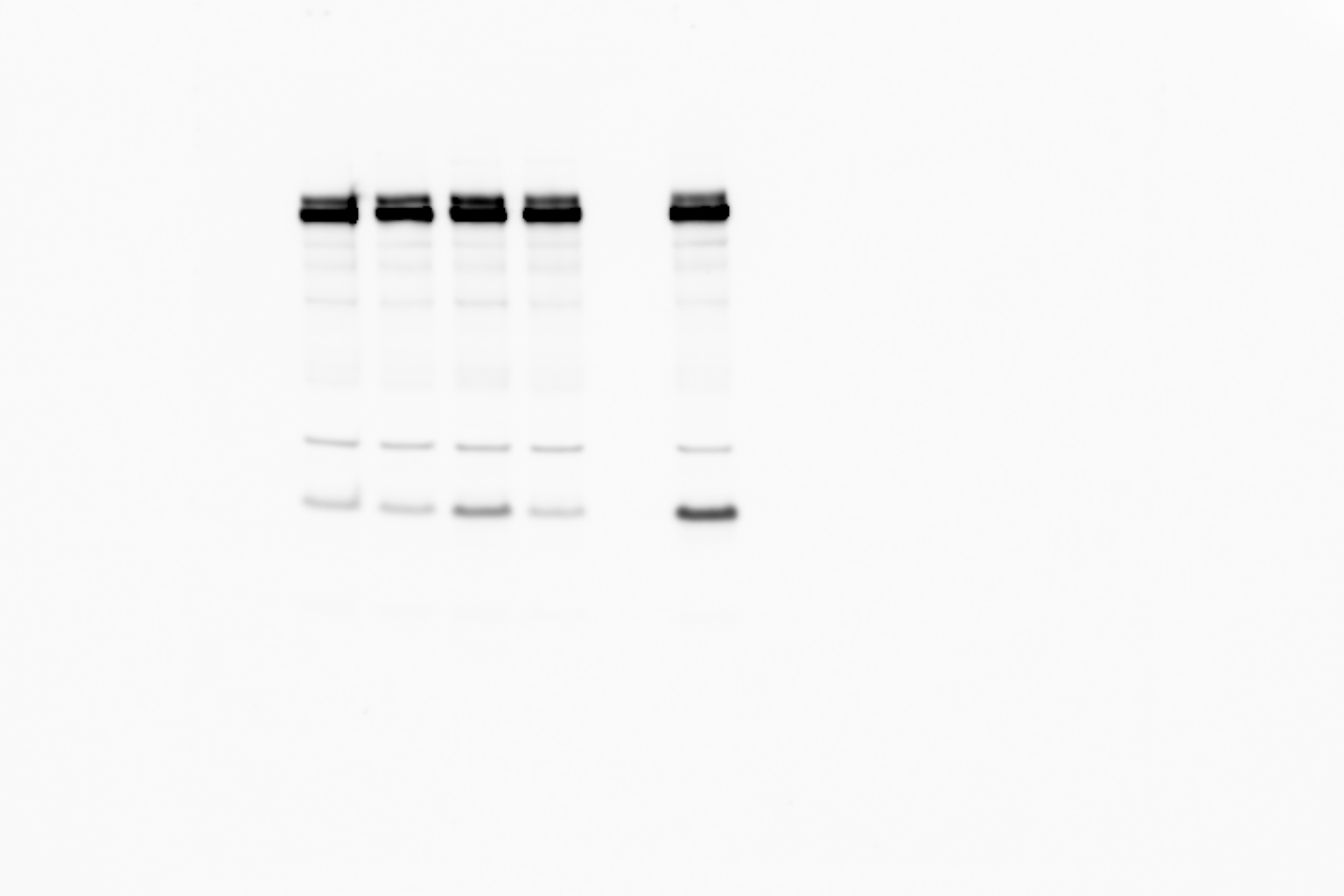

Supplement: Figure 8—source data 1. [file elife-90690-fig8-data1.zip › Figure 8 Source Data 3_org.gel]

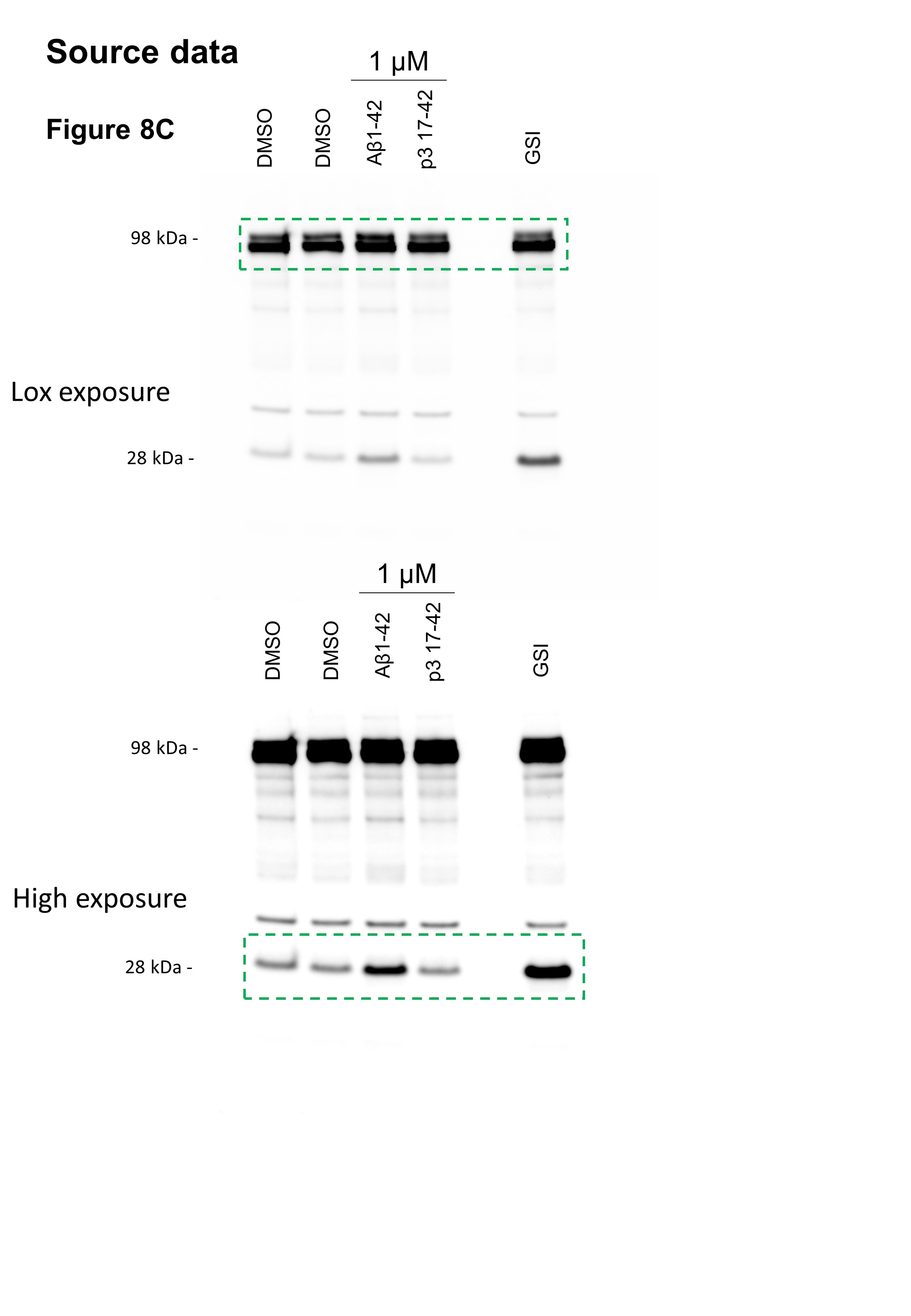

Supplement: Figure 8—source data 1. [file elife-90690-fig8-data1.zip › Figure 8 Source Data 3_panel C.JPG]

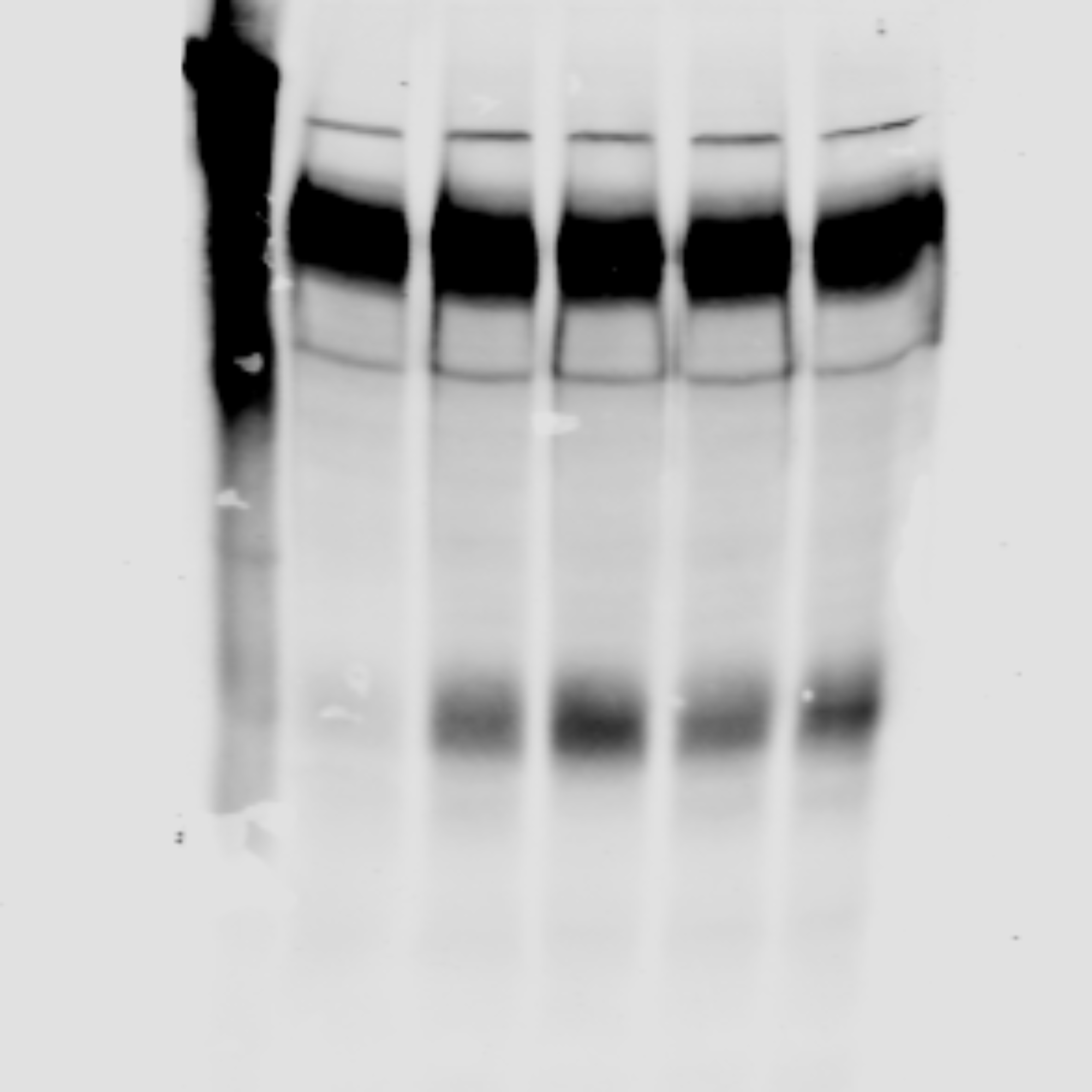

Supplement: Figure 9—source data 1. [file elife-90690-fig9-data1.zip › Figure 9 Source Data 1_org 1.tif]

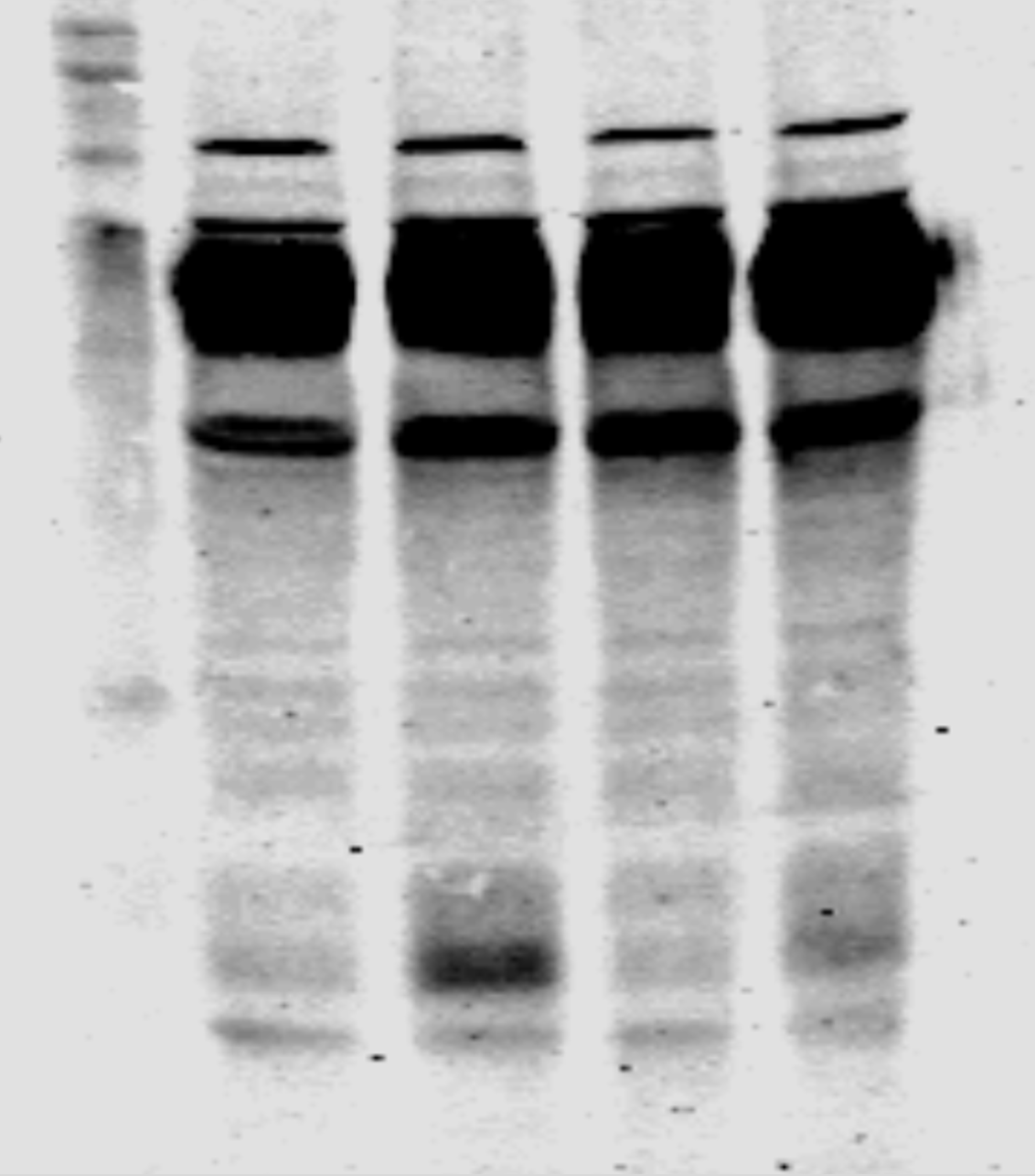

Supplement: Figure 9—source data 1. [file elife-90690-fig9-data1.zip › Figure 9 Source Data 1_org 2.tiff]

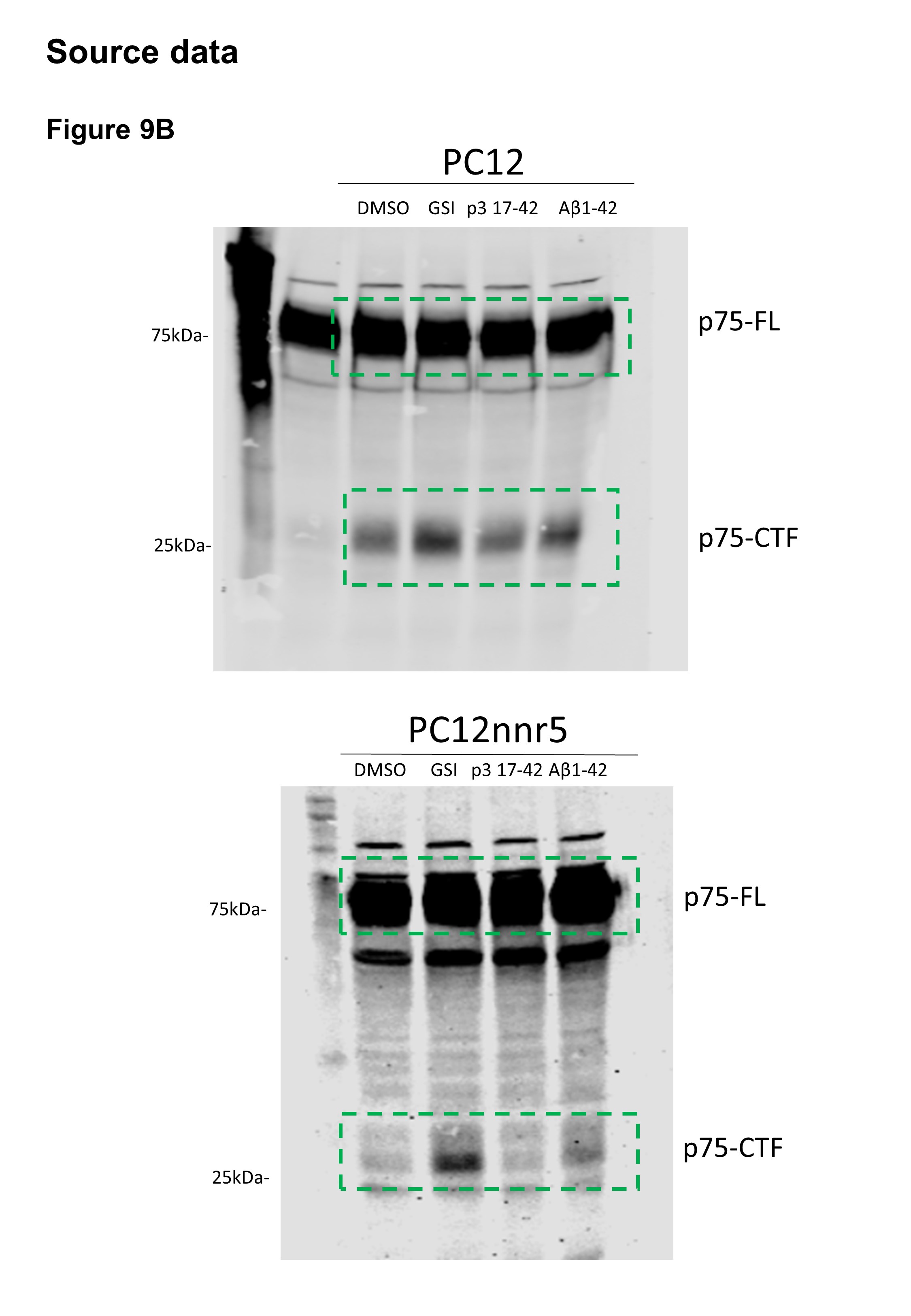

Supplement: Figure 9—source data 1. [file elife-90690-fig9-data1.zip › Figure 9 Source Data 1_panel B.JPG]

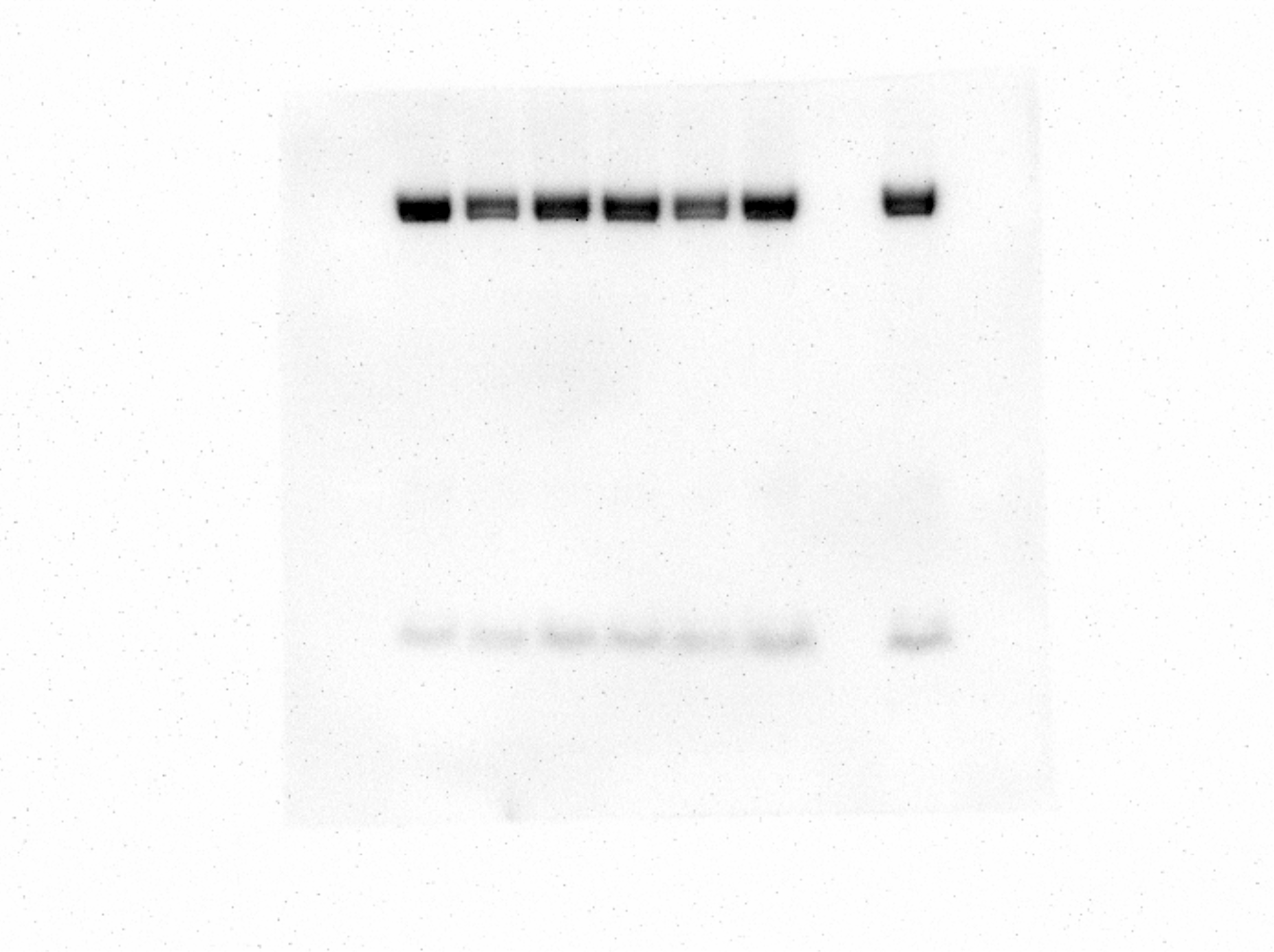

Supplement: Figure 10—source data 1. [file elife-90690-fig10-data1.zip › Figure 10 Source Data 1_org1.tif]

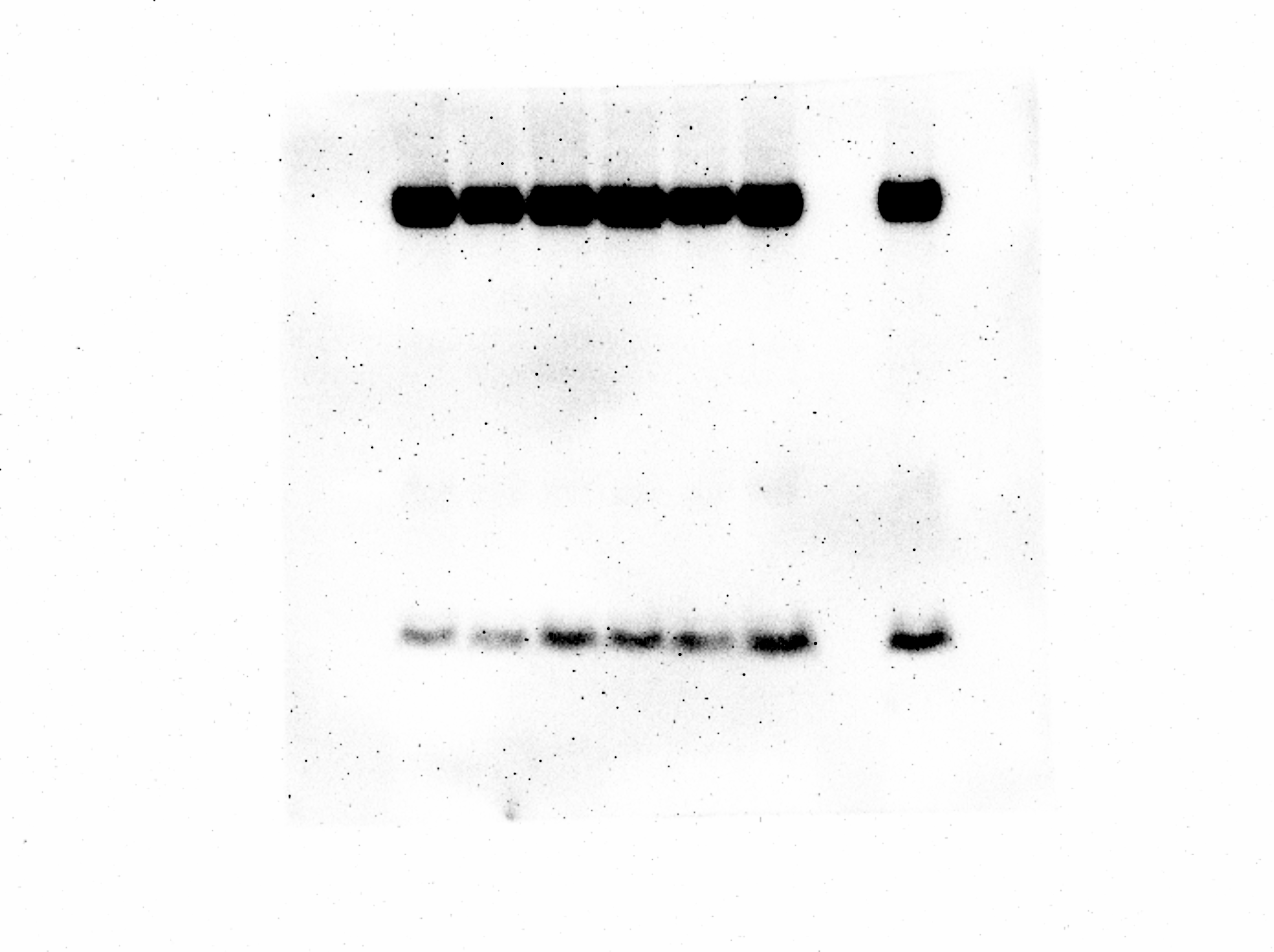

Supplement: Figure 10—source data 1. [file elife-90690-fig10-data1.zip › Figure 10 Source Data 1_org2.tif]

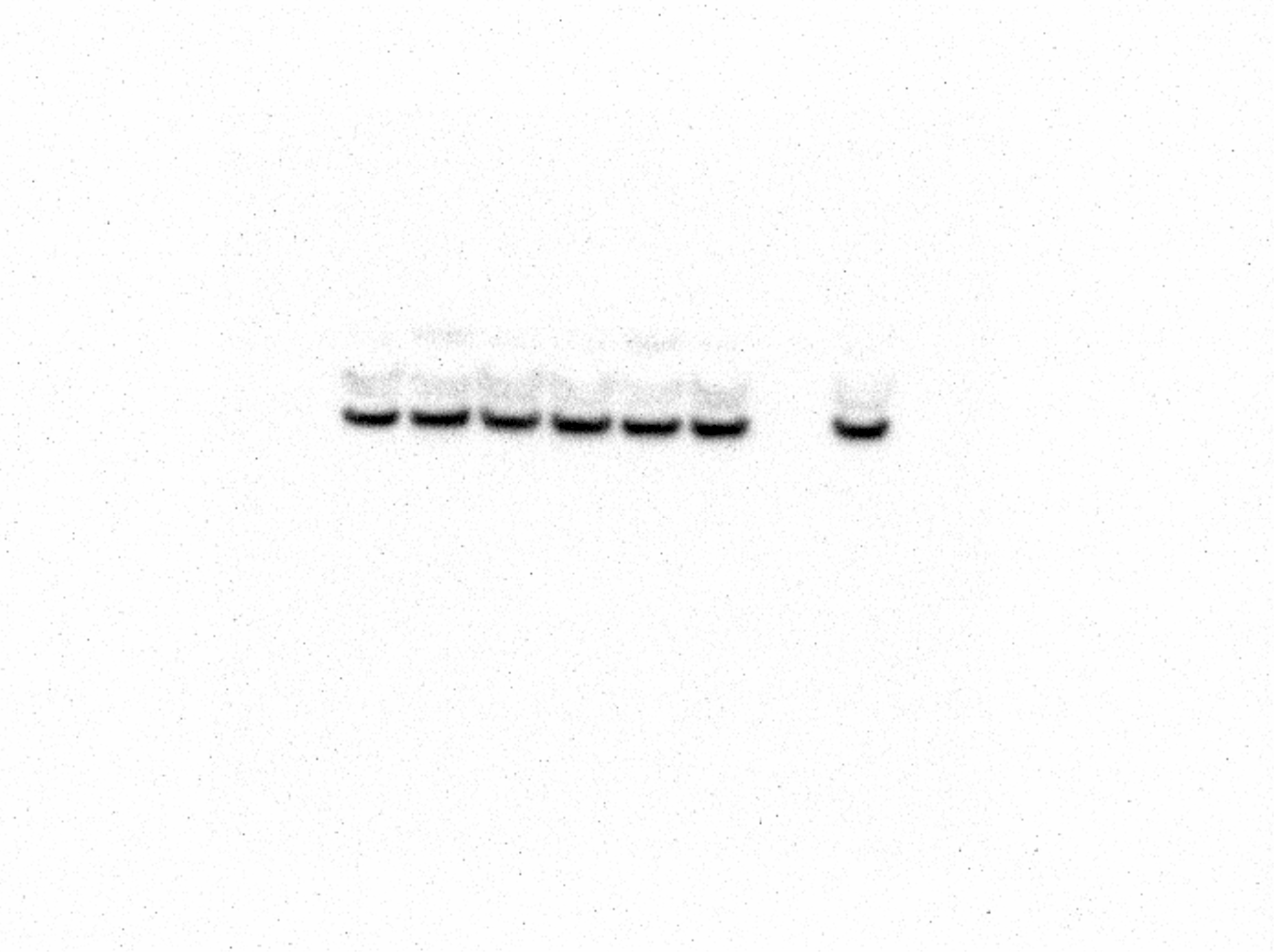

Supplement: Figure 10—source data 1. [file elife-90690-fig10-data1.zip › Figure 10 Source Data 1_org3.tif]

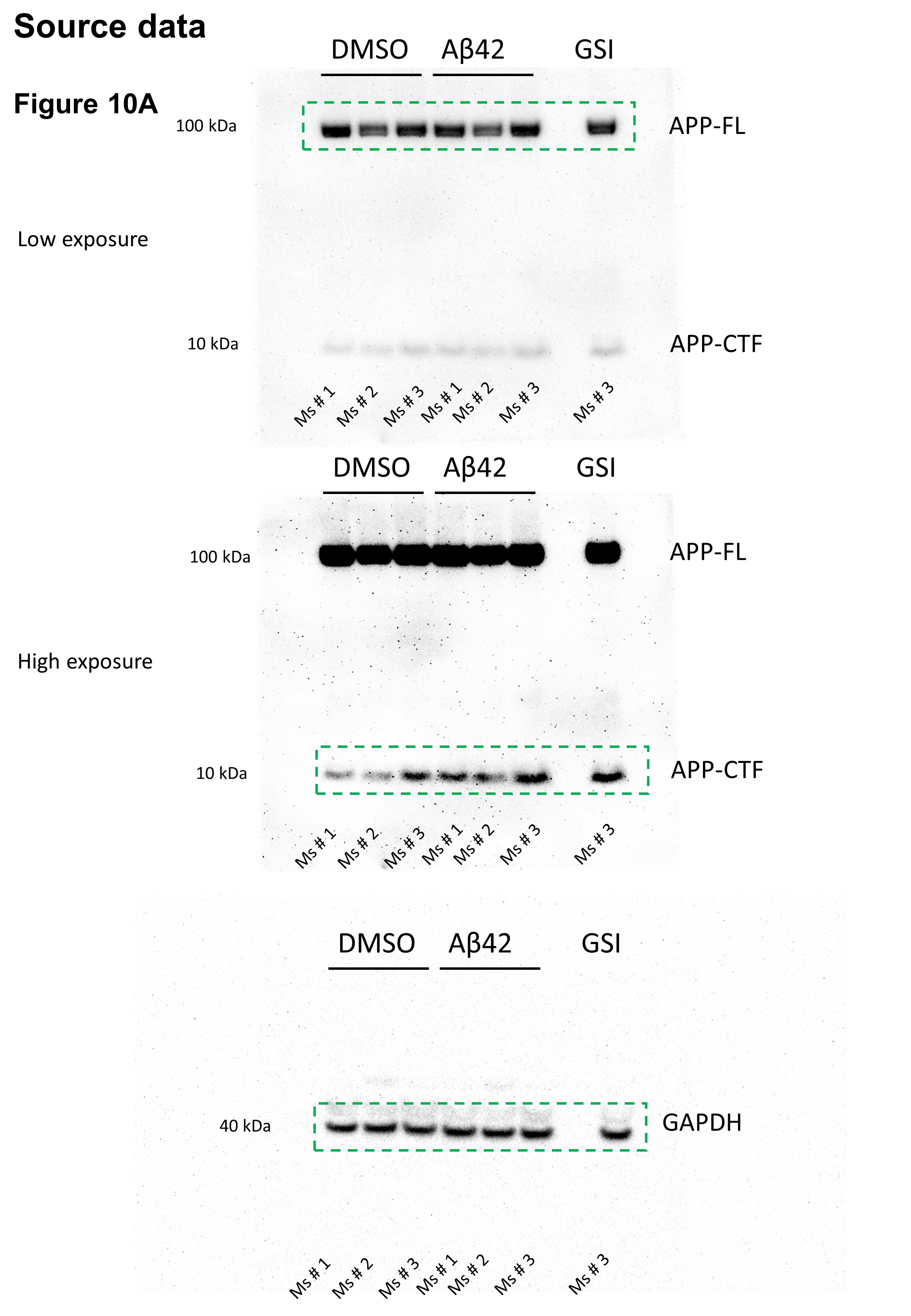

Supplement: Figure 10—source data 1. [file elife-90690-fig10-data1.zip › Figure 10 Source Data 1_panel A.jpg]
