# Supplementary material for: Alzheimer’s disease linked Aβ42 exerts product feedback inhibition on γ-secretase impairing downstream cell signaling
Source: eLife. 2024 Jul 19;12:RP90690. doi: 10.7554/eLife.90690 (PMC11259434; doi:10.7554/eLife.90690)
Supplement: Supplementary file 1. — (a) Table summarizing IC50s in cell-free detergent-based γ-secretase activity assays for selected Amyloid β (Aβ) peptides. (b) Table summarizing demographics of the human post-mortem brain tissue. [file elife-90690-supp1.docx]

**Supplementary File 1**

**Supplementary File 1a. Summary of IC50s in cell-free detergent-based γ-secretase activity assays for selected Aβ peptides**

|  |  | **IC50 [nM]** | |
| --- | --- | --- | --- |
|  |  | **Substrate: 0.4 μM** | **Substrate: 1.2 μM** |
| **AICD** | **Aβ1-42** | 1259 (N=9) | 3565 (N=5) |
|  | **Aβ11-42** | 2218 (N=3) | 4685 (N=4) |
|  | **p3 17-42** | N/A | N/A |
|  | **murine**  **Aβ1-42** | N/A | N/A |
| **NICD** | **Aβ1-42** | 609 (N=3) | 1564 (N=3) |

**Supplementary File 1b. Demographics of the human post-mortem brain tissue**

| **Origin** | **Category** | **Case #** | **Sex** | **Age** | **PMI (h)** | **Clinical diagnosis** | **Additional comments** | **Braak stage** | ***APOEε*X/*ε*X status (X = 2, 3, and/or 4)** |
| --- | --- | --- | --- | --- | --- | --- | --- | --- | --- |
| ADRC-UCI | ND | 19-13 | F | 87 | 3.83 | Normal |  | III | 2/3 |
| ADRC-UCI | ND | 25-01 | M | 83 | 1.8 | Normal |  | II | 3/3 |
| ADRC-UCSD | ND | 5687 | M | 84 | 8 | Normal |  | I | 3/3 |
| ADRC-UCSD | ND | 5783 | M | 84 | 36 | Normal |  | II | 2/3 |
| ADRC-UCSD | ND | 5844 | F | 96 | 6 | Normal |  | II | 3/3 |
| ADRC-UCSD | ND | 5919 | F | 75 |  | Normal | Arteriosclerosis | I | 2/3 |
| ADRC-UCSD | AD | 5401 | M | 81 | 8 | Alzheimer's disease | Amyloid angiopathy | IV | 4/4 |
| ADRC-UCSD | AD | 5620 | M | 86 | 8 | Alzheimer's disease |  | IV | 3/3 |
| ADRC-UCSD | AD | 5835 | F | 85 | 30 | Alzheimer's disease | Lewy body disease- neocortical (diffuse) type | III | 3/3 |
| ADRC-UCSD | AD | 5851 | M | 71 | -4 | Alzheimer's disease | Lewy body disease- neocortical (diffuse) type | IV | 3/4 |
| ADRC-UCSD | AD | 5881 | F | 88 | -4 | Alzheimer's disease | Progressive supranuclear palsy, TDP-43 proteinopathy | III | 3/3 |
| ADRC-UCSD | AD | 5901 | F | 87 | -4 | Alzheimer's disease | Amyloid angiopathy, Lewy body pathology, amygdala predominant type | IV | 3/3 |

**Abbreviations: ADRC, Alzheimer's Disease Research Center; UCI, University of California Irvine; UCSD, University of California San Diego; ND, Non-demented control; AD, Alzheimer's disease; PMI, post-mortem interval. Specimens and their diagnoses were provided by the brain banks at respective ADRCs.**
